# The isolation and characterization of two *Stenotrophomonas maltophilia* bacteriophages capable of cross-taxonomic order infectivity

**DOI:** 10.1186/s12864-015-1848-y

**Published:** 2015-09-03

**Authors:** Danielle L. Peters, Karlene H. Lynch, Paul Stothard, Jonathan J. Dennis

**Affiliations:** Department of Biological Sciences, 6-065 Centennial Centre for Interdisciplinary Science, University of Alberta, Edmonton, AB T6G 2E9 Canada; Department of Agricultural, Food and Nutritional Science, University of Alberta, 1400 College Plaza, Edmonton, AB T6G 2C8 Canada

**Keywords:** *Stenotrophomonas maltophilia*, Bacteriophage, Phage, DNA, Genomics, Phage genome, Delayed lysis, Broad-host-range

## Abstract

**Background:**

A rapid worldwide increase in the number of human infections caused by the extremely antibiotic resistant bacterium *Stenotrophomonas maltophilia* is prompting alarm. One potential treatment solution to the current antibiotic resistance dilemma is “phage therapy”, the clinical application of bacteriophages to selectively kill bacteria.

**Results:**

Towards that end, phages DLP1 and DLP2 (vB_SmaS-DLP_1 and vB_SmaS-DLP_2, respectively) were isolated against *S. maltophilia* strain D1585. Host range analysis for each phage was conducted using 27 clinical *S. maltophilia* isolates and 11 *Pseudomonas aeruginosa* strains. Both phages exhibit unusually broad host ranges capable of infecting bacteria across taxonomic orders. Transmission electron microscopy of the phage DLP1 and DLP2 morphology reveals that they belong to the *Siphoviridae* family of bacteriophages. Restriction fragment length polymorphism analysis and complete genome sequencing and analysis indicates that phages DLP1 and DLP2 are closely related but different phages, sharing 96.7 % identity over 97.2 % of their genomes. These two phages are also related to *P. aeruginosa* phages vB_Pae-Kakheti_25 (PA25), PA73, and vB_PaeS_SCH_Ab26 (Ab26) and more distantly related to *Burkholderia cepacia* complex phage KL1, which together make up a taxonomic sub-family. Phages DLP1 and DLP2 exhibited significant differences in host ranges and growth kinetics.

**Conclusions:**

The isolation and characterization of phages able to infect two completely different species of bacteria is an exciting discovery, as phages typically can only infect related bacterial species, and rarely infect bacteria across taxonomic families, let alone across taxonomic orders.

**Electronic supplementary material:**

The online version of this article (doi:10.1186/s12864-015-1848-y) contains supplementary material, which is available to authorized users.

## Background

The rise in antibiotic resistance amongst bacterial pathogens around the world is causing alarm, with the possibility of a “post-antibiotic era” in the 21st century [1]. One broadly antibiotic-resistant bacterium that is increasing in prevalence in nosocomial and community-acquired infections is *Stenotrophomonas maltophilia*. Some of the infections associated with *S. maltophilia* include pneumonia, bacteremia, meningitis, endocarditis, catheter-related bacteremia/septicemia and acute exacerbations in patients with cystic fibrosis and chronic obstructive pulmonary disease [2, 3]. Preventing infections caused by this bacterium is difficult, as *S. maltophilia* is ubiquitous in the environment and can be easily spread in nosocomial settings by health care providers and cough-generated aerosols [2, 4]. Treatment of *S. maltophilia* infections is problematic due to its innate resistance to a broad array of antibiotics such as trimethoprim/sulfamethoxazole, β-lactams, macrolides, cephalosporins, fluoroquinolones, aminoglycosides, carbapenems, chloramphenicol, tetracyclines and polymyxin. New treatment strategies are thus required in order to successfully combat this extremely drug-resistant bacterium.

One promising treatment strategy is the clinical application of bacteriophages, also known as phage therapy, to selectively kill infecting bacteria [5–10]. Phage therapy has been used for nearly a century in some Eastern European countries, but was largely abandoned in the West during the 1940s due to the advent of broad-spectrum antibiotics. However, with the rise in antibiotic resistance threatening the traditionally effective treatment of bacterial infections, rigorous research into the efficacious use of phage therapy has been renewed. Recent studies utilizing phage therapy in animal models [11–17] and human clinical trials [18–20] have shown that phages can be a successful treatment option. In order to obtain FDA approval for the clinical use of phages, proof is required to show that phage genomes do not encode toxins or other undesirable genes that could potentially enhance bacterial virulence [21]. Therefore, all *S. maltophilia*-specific phages that are to be considered for use in a phage therapy strategy must be fully characterized through complete genome sequencing and analysis to ensure they are safe for use in human clinical trials.

Recent research has led to the isolation and characterization of several different *S. maltophilia* phages, including a jumbo phage phiSMA5 (with a genome of approximately 250 kb in length, [22]), a filamentous phage phiSMA9 with a genome size of 6.9 kb encoding only seven genes, but one of these being a zot toxin [23], a virulent phage Smp14 that exhibits homology to phage T4 [24], a lytic phage IME13 with an unusually large burst size [25], a T7-like phage IME15 specific to *S. maltophilia* [26], a P2-like phage Smp131 whose genome exhibits sequence homology to prophages in *Xanthomonas* species [27], and three other novel, small filamentous phages phiSMA 6, phiSMA7 and phiSHP1 [28, 29]. Twenty-two phages specific for different *Stenotrophomonas* species, including the well-characterized temperate phage S1, have also recently been isolated [30]. Additional pertinent research has shown that non-interactive *Lactococcus* phages can easily penetrate the biofilms produced by *S. maltophilia* [31, 32]. Here we describe the isolation and characterization of two novel *S. maltophilia* phages DLP1 and DLP2. These phages are related to three previously characterized *Pseudomonas aeruginosa* phages and have the unusual characteristic of cross-taxonomic order infectivity, the ability to use strains of both *S. maltophilia* and *P. aeruginosa* as hosts for phage propagation.

## Results and discussion

### Isolation, host range and morphology

Using *S. maltophilia* strain D1585, phages DLP1 and DLP2 were isolated from Red Deer River sediment and soil planted with blue flax (*Linum lewisii*), respectively. In contrast to the characterized *S. maltophilia* phages isolated from clinical settings, sewage samples and lysogenic bacteria [22–30], DLP1 and DLP2 are the first phages to be isolated from sediment and soil.

**Fig. 1:**
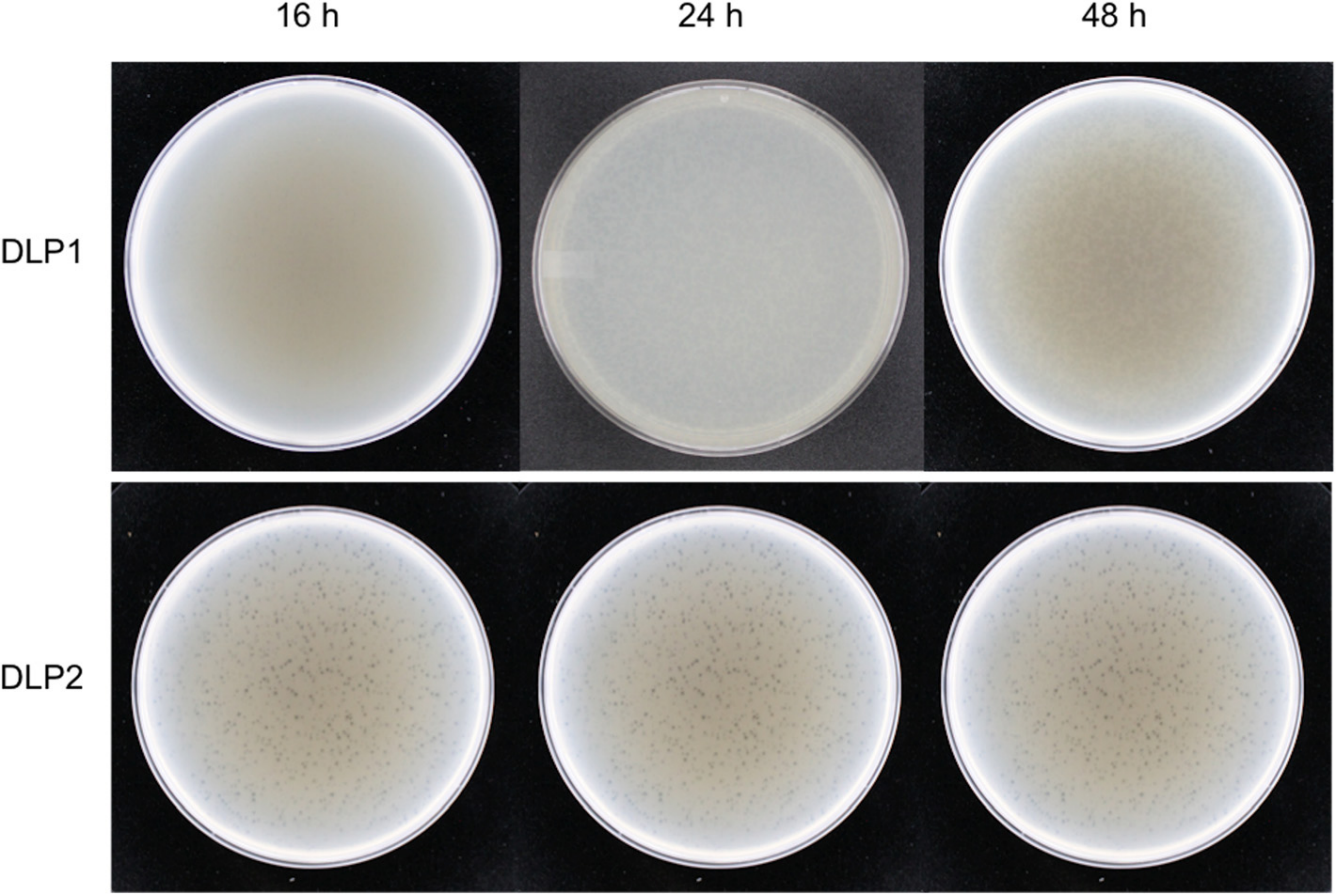
Development and morphology of DLP1 and DLP2 plaques. Phages were plated in half-strength Luria-Bertani (½ LB) agar overlays with 16 h liquid culture of *Stenotrophomonas maltophilia* D1585. Plates were incubated at 30 °C and photographed at 16, 24 and 48 h. Turbid DLP1 plaques were difficult to visualize until after 24 h of growth, whereas clear, well-defined DLP2 plaques were observed after 16 h

Phage DLP1 exhibits a unique plaque development that was previously identified in phages KL1 and AH2 that target bacteria of the *Burkholderia cepacia* complex [33]. As with KL1 and AH2, stocks of DLP1 can be concentrated (up to 10^10^ plaque forming units [PFU]/ml), but use of such high titre stocks results in plates with no plaques. Instead, when lower titres (10^7^ PFU/ml or less) are used, and the plates are incubated at 30 °C for at least 24 h, DLP1 plaque development occurs (Fig. 1). Individual plaques for DLP1 are turbid with no distinct boarders and a diameter of 0.4-1 mm, averaging 0.7 mm. This contrasts the plaque development of phage DLP2, which produces clearing at high titres and clearly defined plaques at lower titres following 16 h incubation at 30 °C (Fig. 1). Plaque sizes for DLP2 are clear with distinct boarders and a diameter 0.2-0.8 mm, averaging 0.4 mm.

**Fig. 2:**
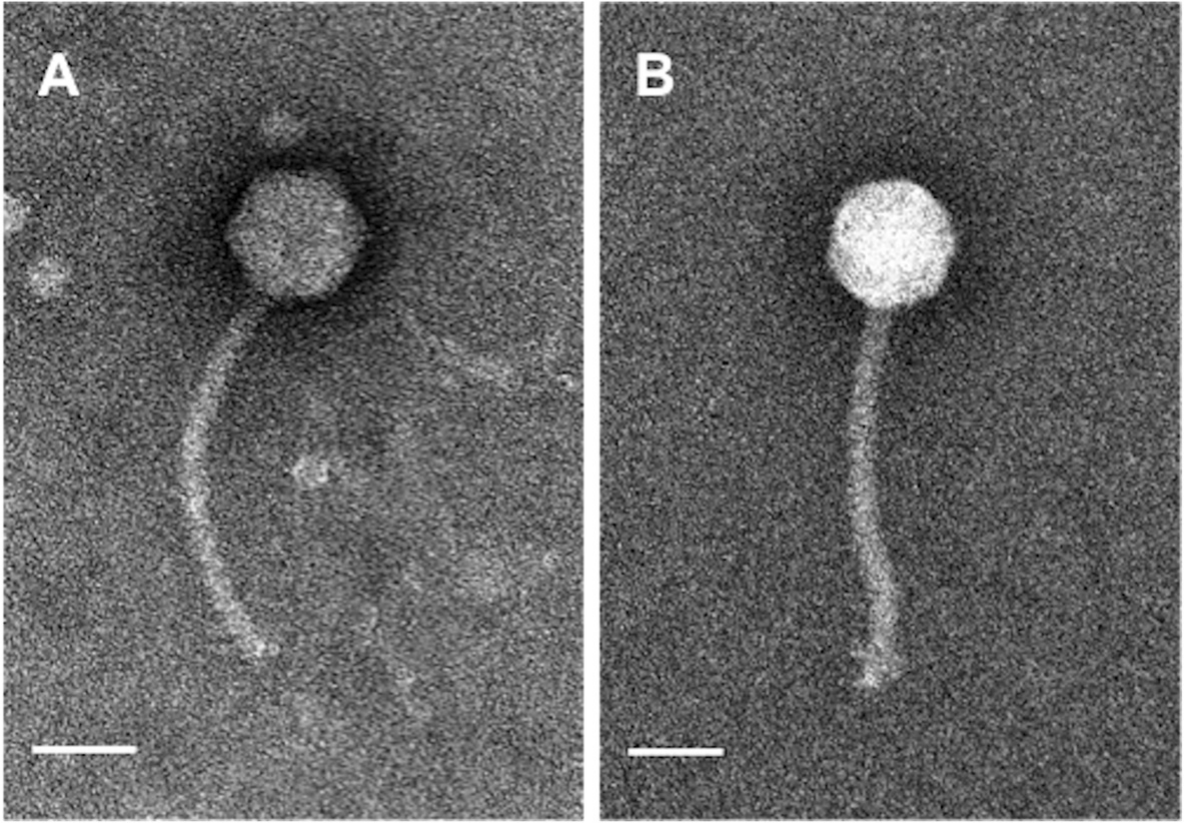
DLP1 (**a**) and DLP2 (**b**) phage morphology. Phages were stained with 4 % uranyl acetate and visualized at 180,000-fold magnification by transmission electron microscopy. Scale bars represent 50 nm. Both *Siphoviridae* family phages were of similar size, although the tail of DLP1 (175 nm) was shorter than that of DLP2 (205 nm)

DLP1 and DLP2 are classified in the order *Caudovirales* and the family *Siphoviridae* due to their morphological characteristics observed using electron microscopy. The DLP1 phage has a long, non-contractile tail of approximately 175 nm in length and a capsid size of approximately 70 nm in diameter (Fig. 2a). Phage DLP2 is larger, with a non-contractile tail of approximately 205 nm and a capsid size of approximately 70 nm in diameter (Fig. 2b).

**Fig. 3:**
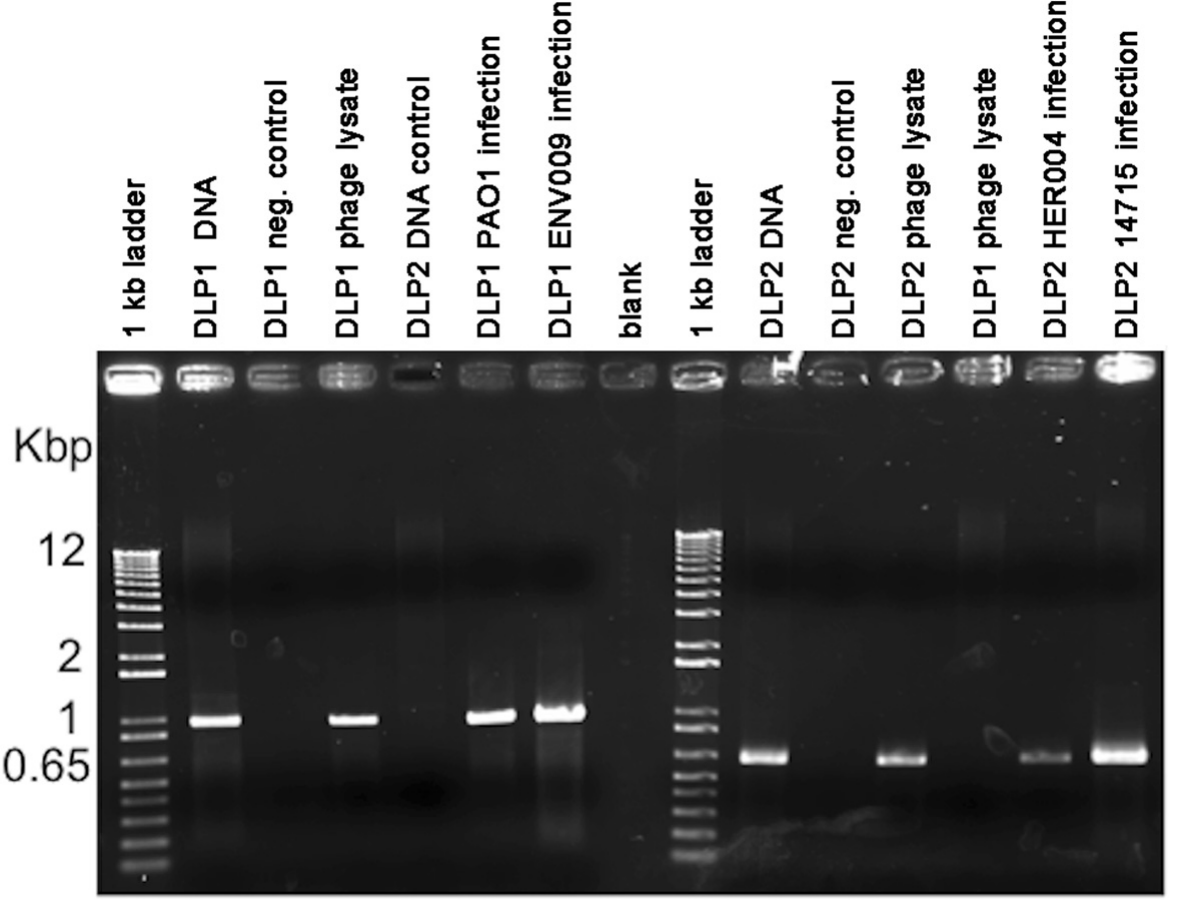
PCR confirmation of *P. aeruginosa* infections by DLP1 and DLP2. Lanes 1 and 9: 1 Kb Plus DNA ladder (Invitrogen), lane 2: DLP1 phage DNA, lane 3: DLP1 negative control, lane 4: DLP1 phage lysate, lane 5: DLP2 phage DNA, lane 6: DLP1 phage lysate from PA01 infection, lane 7: DLP1 phage lysate from ENV009 infection, lane 8: blank, lane 10: DLP2 phage DNA, lane 11: DLP2 negative control, lane 12: DLP2 phage lysate, lane 13: DLP1 phage lysate, lane 14: DLP2 phage lysate from HER004 infection, lane 15: DLP2 phage lysate from 14,715 infection. The size of the markers (in Kbp) is shown on the left

Both DLP1 and DLP2 have a moderate host range within the *S. maltophilia* strains tested, with the ability to infect eight and nine out of 27 strains, respectively (Table 1). Both phages also have a unique ability to infect across bacterial taxonomic orders, with each phage capable of infecting two separate *P. aeruginosa* strains each (Table 2, Additional file: 1Table S1). This is an interesting finding, as bacteriophages are typically thought to be relatively species specific. However, there are examples of bacteriophages that have been shown to lyse bacteria of different genera. For example, some phages originally discovered to infect one genus of Cyanobacteria, have also been shown to be able to lyse other Cyanobacteria genera [34–36]. Confirmation of successful DLP1 and DLP2 infection and lysis of *P. aeruginosa* strains was confirmed with the use of PCR (Fig. 3).

**Table 1:** Host range analysis of DLP1 and DLP2 against *Stenotrophomonas maltophilia* clinical isolates

*S. maltophilia* strain	Phage	
	DLP1	DLP2
101	+	+
102	–	+
103	+	+
152	–	–
155	–	–
174	–	–
176	–	–
213	+	+
214	–	–
217	–	–
218	+	–
219	–	–
230	–	–
236	+	+
242	+	+
249	+	+
278	–	–
280	–	++++
282	–	–
287	–	–
446	–	–
667	–	–
D1585^a^	++++	++++
D1571^a^	–	–
D1614^a^	–	–
D1576^a^	–	–
D1568^a^	–	–

–, No sensitivity to phage; +, plaques at 10^−2^; ++, clearing at 10^−2^; +++, plaques at 10^−4^; ++++, plaques at 10^−6^

^a^Isolates from the Canadian *Burkholderia cepacia* complex Research Referral Repository

**Table 2:** Host range analysis of DLP1 and DLP2 against *Pseudomonas aeruginosa* strains

*P. aeruginosa* strain	Phage	
	DLP1	DLP2
PA01	++	–
HER1004	–	+++
HER1012	–	–
14,715	–	++
Utah3	–	–
Utah4	–	–
14,655	_	–
6106	_	–
pSHU-OTE	–	–
D1606D^a^	–	–
D1615C^a^	–	–
D1619M^a^	–	–
D1620E^a^	–	–
D1623C^a^	–	–
ENV003^a^	–	–
ENV009^a^	+++	–
FC0507^a^	–	–
R285	–	–
14,672	–	–

–, No sensitivity to phage; +, plaques at 10^−2^; ++, clearing at 10^−2^; +++, plaques at 10^−4^; ++++, plaques at 10^−6^

^a^Isolates from the Canadian *Burkholderia cepacia* complex Research Referral Repository

### Genome characterization

**Fig. 4:**
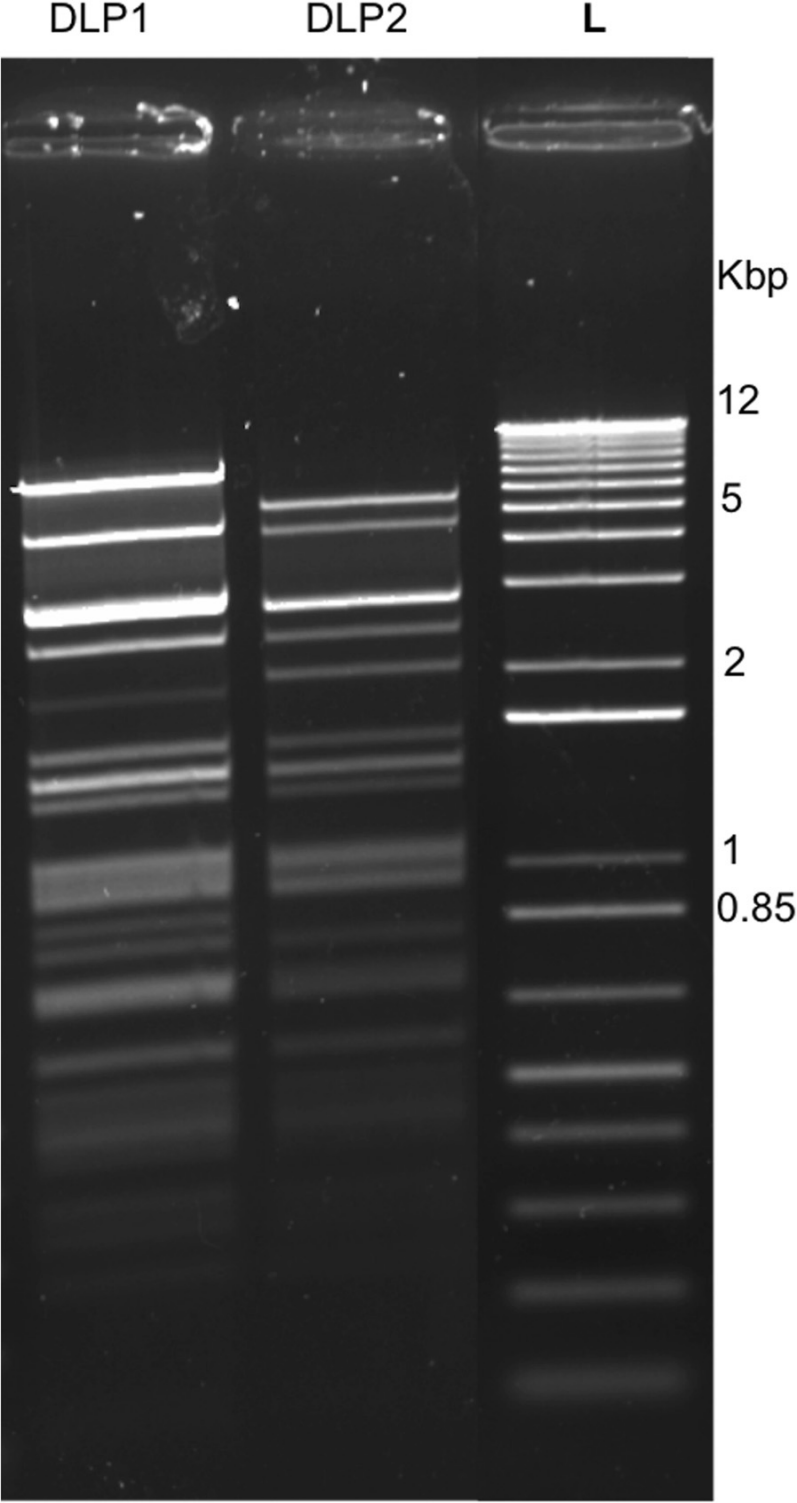
Restriction fragment length polymorphism of DLP1 and DLP2 genomic DNA. 1 μg of phage genomic DNA was digested 5 min with EcoRI and separated on a 1 % agarose gel. L: 1 Kb Plus DNA Ladder (Invitrogen). Several differences in banding pattern between the genomic DNAs isolated from the two phages is apparent

**Fig. 5:**
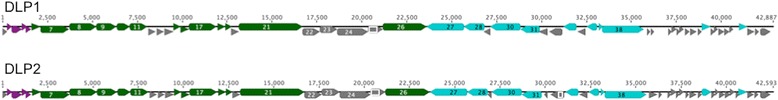
Genome maps of DLP1 and DLP2. The scale (in kbp) is shown above the DLP1 and DLP2 genomic maps. The assigned function of the predicted proteins encoded by each open reading frame is as follows: grey - unknown function; purple - lysis; green - virion morphogenesis; blue - DNA replication/repair. Numbers within the larger ORFs relate to gene product number

**Fig. 6:**
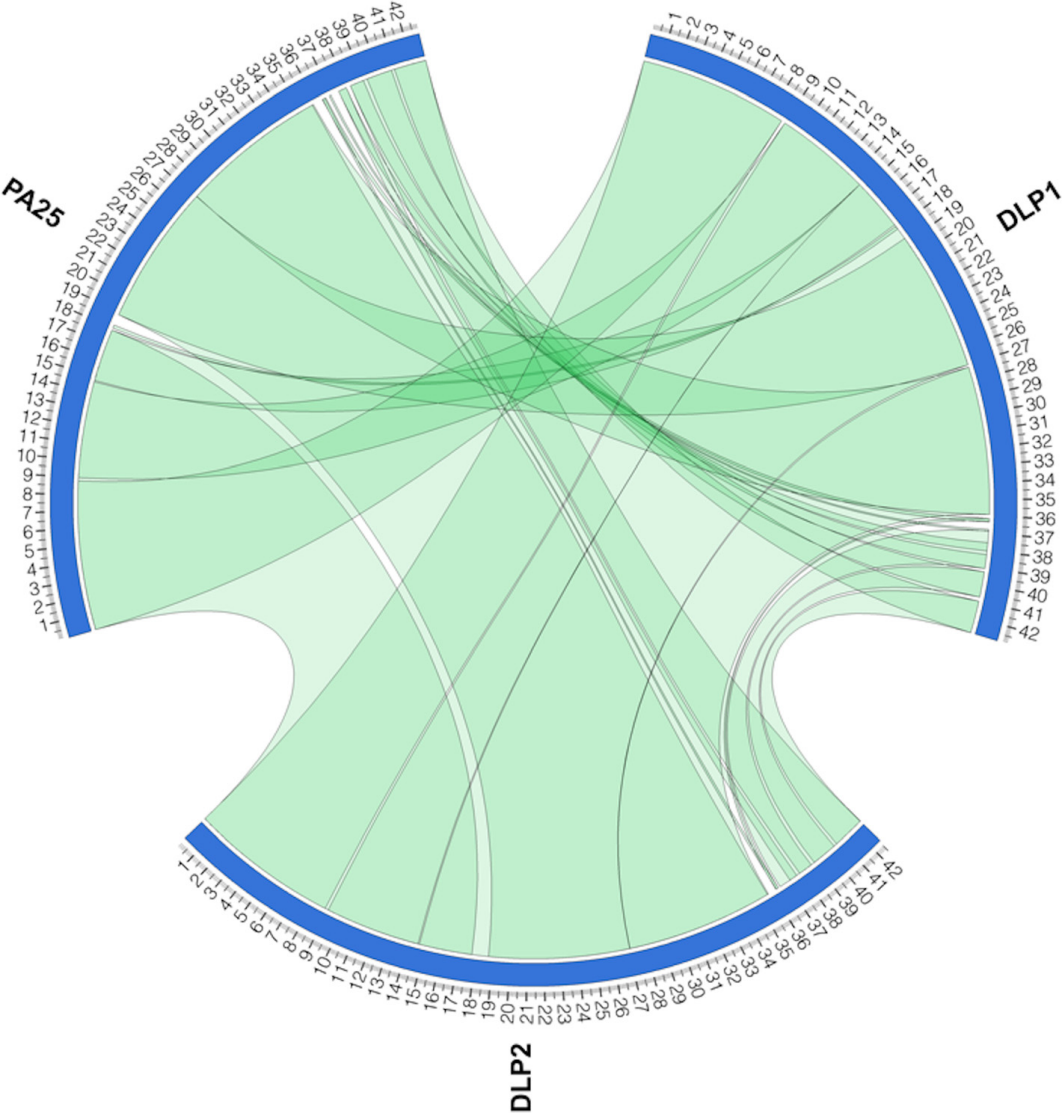
Circos plot of genomes from phages DLP1, DLP2 and vB_Pae-Kakheti25 (PA25) NUCmer comparisons. Green ribbon indicates regions of similarity between the three genomes at the nucleotide level. The scale (in kbp) is shown on the periphery of the plot for each phage. NUCmer parameters: breaklen = 200, maxgap = 90, mincluster = 65, minmatch = 20

Genomic analysis of phages DLP1 and DLP2 reveals they are closely related phages. Initially, a comparison of restriction fragment length polymorphisms (RFLPs) of DLP1 and DLP2 EcoRI-digested genomic DNA shows similar banding patterns with slight band differences between 0.85-1, 2-3 and 5 kbp (Fig. 4). DLP1 and DLP2 similarity was confirmed by the results of the whole genome sequencing using the Illumina platform (discussed below). A genome map for DLP1 and DLP2 (Fig. 5) shows the modular similarity of the two phages, as well as their genetic similarity with respect to their genes and genome sizes. However, complete genome sequencing also demonstrates the crudeness of RFLP analysis. The DLP1 genome contains 31 EcoRI sites, whereas the DLP2 genome possesses 32 EcoRI sites. Phage DLP1 possesses five DNA insertions of 29 bp in EcoRI fragment 6869-9910, 40 bp in fragment 9910-10,628, 50 bp in fragment 12,987-13,729, 129 bp in fragment 24,500-27,657 and 118 bp in fragment 34,709-39,879. Phage DLP2 possesses three DNA insertions of 40 bp in EcoRI fragment 10,559-11,971, 87 bp in fragment 14,003-14,999, and 5 bp in fragment 29,984-31,617. In addition, phage DLP2 has an extra EcoRI site at base pair 3345 due to a point mutation. Phages DLP1 and DLP2 were found to be 96.7 % identical over 97.2 % of their genomes. However, this comparison still denotes considerable variation between the two phage genomic sequences. A BLASTN comparison indicates that the two genomes share 40,317 identical base pairs out of 41,687 aligned base pairs (1200 base pairs unaligned), with 166 gaps. The similarity of DLP1 and DLP2 to each other, and to their closest relative *Pseudomonas* phage vB_Pae-Kakheti25 (informally PA25), is illustrated in Fig. 6, a Circos plot of a NUCmer comparison of the three phages.

The DLP1 genome is 42,887 base pairs (bp) in length, with a GC content of 53.7 %. DLP1 is predicted to encode 57 proteins with the most common start codon being ATG, although a TTG start codon is used for gp19, gp37 and gp41 (Table 3, Fig. 5). Besides phage DLP2, phage DLP1 is most closely related to the siphovirus *Pseudomonas* phage vB_Pae-Kakheti25 (PA25) (Fig. 6) [37]. DLP1 and PA25 phages are similar with respect to genome length (42,844 bp for PA25 and 42,887 bp for DLP1), GC content (53.7 % for PA25 and DLP1) and predicted number of proteins (58 for PA25 and 57 for DLP1) [37]. BLASTN comparison of DLP1 and PA25 shows a 98 % identity over 94 % of the genome. The genome of DLP1 also shows high similarity to phages PA73 (98 % identity over 92 % of the genome) [38] and vB_PaeS_SCH_Ab26 (Ab26) (96 % identity over 92 % of the genome) [37]. Again, this represents a significant amount of genetic variation, with 145 gaps required to complete the genomic alignment with PA25, 144 gaps required to align PA73, and 220 gaps required to align Ab26, suggesting that although these phages belong to the same family, they are not merely variants of one another. The predicted proteins of DLP1 are similar to those found in phages PA25, PA73 and Ab26; though DLP1 proteins gp32, gp45 and gp48 are unique in that they are not similar to any proteins found in PA25, PA73 and Ab26 (Table 3). The DLP1 protein gp32 is related to gp055 of the *Erwinia* phage vB_EamP-S6. The gp45 protein is related to the hypothetical protein X805_23910 of *Sphaerotilus natans* subsp. *natans* DSM 6575, which is a filamentous bacterium known to contribute to the stability of *Pseudomonas* sp. colonies at low concentrations [39]. The Vsr endonuclease encoded by gp48 is most homologous to a gene found in the *Burkholderia* phage KL1 [33].

**Table 3:** Bacteriophage DLP1 genome annotation

Gene	Start	End	Putative function	Strand	Length (AA)	Closest relative	Percent identity	Source	GenBank accession number
1	1	255	Hypothetical protein	+	84	ORF001	100	PA73	YP_001293408.1
2	252	518	Holin	+	88	Holin	99	PA25	YP_006299866.1
3	511	1,056	Endolysin	+	181	Endolysin	98	PA25	YP_006299867.1
4	1068	1373	Rz	+	101	Rz	99	Ab26	YP_009044338.1
5	1288	1569	Rz1	+	93	Rz1	100	PA25	YP_006299869.1
6	1627	2115	Small terminase	+	162	Small terminase	99	PA25	YP_006299870.1
7	2096	3691	Large terminase	+	531	Large terminase	100	Ab26	YP_001293413.1
8	3705	5210	Portal protein	+	501	Portal protein	98	Ab26	YP_009044342.1
9	5222	6316	F-like head morphogenesis protein	+	364	ORF008	100	PA73	YP_001293415.1
10	6353	7072	Scaffold protein	+	239	Scaffold protein	100	PA25	YP_006299874.1
11	7075	8052	Major capsid protein	+	325	ORF010	99	PA73	YP_001293417.1
12	8122	8526	Hypothetical protein	+	134	ORF011	100	PA73	YP_001293418.1
13	8592	8993	Hypothetical protein	+	133	ORF12	71	Ab26	YP_009044347.1
14	9005	9523	Hypothetical protein	+	172	Hypothetical protein	92	Ab26	YP_009044348.1
15	9527	9907	Head-tail joining protein	+	126	Hypothetical protein	98	PA25	YP_006299879.1
16	9904	10,359	Minor tail protein	+	151	ORF015	97	PA73	YP_001293422.1
17	10,372	11,907	Major tail tube protein	+	511	Major tail tube protein	99	PA25	YP_006299881.1
18	11,971	12,399	Tail chaperonin	+	142	ORF017	100	PA73	YP_001293424.1
19	12,408	12,764	Tail chaperonin	+	118	Tail chaperonin	100	PA25	YP_006299882.1
20	12,733	13,167	Hypothetical protein	+	144	ORF019	100	PA73	YP_001293426.1
21	13,173	16,708	Tape measure protein	+	1175	Tape measure protein	96	PA25	YP_006299885.1
22	16,701	17,663	Hypothetical protein	+	320	Hypothetical protein	87	PA25	YP_006299886.1
23	17,663	18,628	Hypothetical protein	+	321	Hypothetical protein	64	PA25	YP_006299887.1
24	18,634	20,346	Hypothetical protein	+	570	Hypothetical protein	96	PA25	YP_006299888.1
25	20,346	21,170	Hypothetical protein	+	274	Hypothetical protein	99	PA25	YP_006299889.1
26	21,174	23,615	Central tail hub	+	813	Central Tail Hub	99	PA25	YP_006299890.1
27	23,616	25,667	DNA polymerase	-	683	DNA polymerase	99	PA25	YP_006299891.1
28	25,679	26,821	Replicative clamp	-	380	Replicative clamp	99	PA25	YP_006299892.1
29	26,805	27,161	Hypothetical protein	-	118	ORF028	97	PA73	YP_001293435.1
30	27,166	28,821	DEAD box helicase	-	551	ORF029	100	PA73	YP_001293436.1
31	28,814	29,911	RecB exonuclease	-	365	ORF030	100	PA73	YP_001293437.1
32	29,817	30,344	Hypothetical protein	-	175	gp055	41	EamP-S6^a^	YP_007005791.1
33	30,423	31,169	Hypothetical protein	-	248	Member of the DUF669 phage protein family	99	PA25	YP_006299897.1
34	31,228	31,944	RecA	-	238	RecA	99	Ab26	YP_009044366.1
35	31,999	32,439	Hypothetical protein	-	146	ORF033	99	PA73	YP_001293440.1
36	32,516	33,073	MazG	-	185	MazG	89	PA25	YP_006299900.1
37	33,193	33,399	Transcriptional regulator	+	68	ORF035	100	PA73	YP_001293442.1
38	33,389	35,710	Replicative primase/helicase	+	773	Replicative primase/helicase	99	PA25	YP_006299902.1
39	35,862	36,062	Hypothetical protein	+	66	Hypothetical protein	99	Ab26	YP_009044372.1
40	36,107	36,256	Hypothetical protein	+	49	KAK25_00040	96	PA25	YP_006299904.1
41	37,055	37,234	Hypothetical protein	+	59	Hypothetical protein ORF0038	92	PA73	YP_001293445.1
42	37,231	37,527	Hypothetical protein	+	98	ORF0039	98	PA73	YP_001293446.1
43	37,524	37,703	Hypothetical protein	+	59	Hypothetical protein	90	Ab26	YP_009044375.1
44	37,678	37,920	Hypothetical protein	+	80	Hypothetical protein	96	Ab26	YP_009044376.1
45	38,003	38,224	Hypothetical protein	+	73	X805_23910	56	DSM 6575^b^	KDB52021.1
46	38,271	38,645	Hypothetical protein	+	124	KAK25_00046	99	PA25	YP_006299910.1
47	38,706	38,927	Hypothetical protein	+	73	KAK25_00047	97	PA25	YP_006299911.1
48	38,924	39,415	Vsr endonuclease	+	163	Vsr endonuclease	78	KL1^c^	YP_006560795.1
49	39,403	39,618	Hypothetical protein	+	71	Hypothetical protein	99	Ab26	YP_009044380.1
50	39,615	39,794	Hypothetical protein	+	59	KAK25_00050	95	PA25	YP_006299914.1
51	39,855	40,154	Hypothetical protein	+	99	ORF0045	81	PA73	YP_001293452.1
52	40,171	40,461	Hypothetical protein	+	96	ORF0046	99	PA73	YP_001293453.1
53	40,454	40,687	Hypothetical protein	+	77	KAK25_00053	100	PA25	YP_006299917.1
54	40,983	41,450	dCMP deaminase	+	155	dCMP deaminase	98	PA25	YP_006299919.1
55	41,456	41,839	Hypothetical protein	+	127	ORF0050	100	PA73	YP_001293457.1
56	41,874	42,083	Hypothetical protein	+	69	ORF0051	96	PA73	YP_001293458.1
57	42,167	42,739	Hypothetical protein	+	190	ORF0052	99	PA73	YP_001293459.1

^a^ *Erwinia* phage vB_EamP-S6

^b^ *Sphaerotilus natans* subsp. *natans* DSM 6575

^c^KL1 is *Burkholderia* phage KL1

The DLP2 genome is 42,593 bp in length, with a GC content of 53.7 %. DLP2 is predicted to encode 58 proteins with the most common start codon being ATG, although a TTG start codon is used for gp19 and gp37, and a GTG start codon is used for gp43 and gp55 (Table 4, Fig. 5). Phage DLP2 is also related to *Pseudomonas* phage PA25 (Fig. 6). These two phages are similar with respect to genome length (42,844 bp for PA25 and 42,593 for DLP2), GC content (53.7 % for PA25 and DLP2) and predicted number of proteins (58 for PA25 and DLP2) [40]. BLASTN comparison of the DLP2 and PA25 genomes shows a 97 % identity over 95 % of the genome. The BLASTN results also reveals DLP2 to be similar to *Pseudomonas* phages PA73 (98 % identity over 93 % of genome) [38] and Ab26 (97 % identity over 90 % of the genome) [37]. Phage DLP2 gene content differs from phages PA25, PA73 and Ab26 in predicted proteins gp39 and gp45 (Table 4). DLP2 protein gp39 is most closely related to the uncharacterized protein MAM_066 of the *Serratia* phage ΦMAM1. Similar to DLP1, the DLP2 gp45 protein is related to the hypothetical protein X805_23910 of *Sphaerotilus natans* subsp. *natans* DSM 6575.

**Table 4:** Bacteriophage DLP2 genome annotation

Gene	Start	End	Putative function	Strand	Length (AA)	Homologue	Percent identity	Source	GenBank accession number
1	1	255	Hypothetical protein	+	84	Phage protein found in lysis cassettes	96	PA25	YP_006299865.1
2	252	518	Holin	+	88	Holin	95	PA25	YP_006299866.1
3	511	1056	Endolysin	+	181	ORF003	100	PA73	YP_001293410.1
4	1068	1373	Rz	+	101	Rz	100	Ab26	YP_009044338.1
5	1288	1569	Rz1	+	93	Rz1	100	Ab26	YP_009044339.1
6	1627	2115	Small terminase	+	162	Small terminase	100	PA25	YP_006299870.1
7	2096	3691	Large terminase	+	531	Large terminase	99	Ab26	YP_009044341.1
8	3705	5210	Portal protein	+	501	Portal protein	99	Ab26	YP_009044342.1
9	5222	6316	F-like head morphogenesis protein	+	364	F-like head morphogenesis protein	99	Ab26	YP_009044343.1
10	6353	7072	Scaffold protein	+	239	Scaffold protein	98	PA25	YP_006299874.1
11	7075	8052	Major capsid protein	+	325	ORF010	99	PA73	YP_001293417.1
12	8122	8526	Hypothetical protein	+	134	ORF011	99	PA73	YP_001293418.1
13	8592	8963	Hypothetical protein	+	123	ORF012	98	PA73	YP_001293419.1
14	8976	9494	Hypothetical protein	+	172	Virion protein	100	PA25	YP_006299878.1
15	9498	9878	Head-tail joining protein	+	126	Virion protein	98	Ab26	YP_009044349.1
16	9875	10,330	Minor tail protein	+	151	ORF015	99	PA73	YP_001293422.1
17	10,343	11,878	Major tail tube protein	+	511	Major tail tube protein	99	PA25	YP_006299881.1
18	11,942	12,370	Tail chaperonin	+	142	Tail chaperonin	99	Ab26	YP_009044352.1
19	12,379	12,735	Tail chaperonin	+	114	Tail chaperonin	99	Ab26	YP_009044353.1
20	12,704	13,138	Hypothetical protein	+	144	ORF019	100	PA73	YP_001293426.1
21	13,144	16,711	Tape measure protein	+	1187	Tape measure protein	99	PA25	YP_006299885.1
22	16,709	17,671	Hypothetical protein	+	320	Virion protein	88	PA25	YP_006299886.1
23	17,671	18,636	Hypothetical protein	+	321	Virion protein	64	PA25	YP_006299887.1
24	18,642	20,354	Hypothetical protein	+	570	Virion protein	96	PA25	YP_006299888.1
25	20,354	21,178	Hypothetical protein	+	274	Virion protein	99	PA25	YP_006299889.1
26	21,182	23,623	Central tail hub	+	813	Central tail hub	99	PA25	YP_006299890.1
27	23,624	25,675	DNA polymerase	-	683	DNA polymerase	99	PA25	YP_006299891.1
28	25,687	26,829	Replicative clamp	-	380	Replicative clamp	97	PA25	YP_006299892.1
29	26,813	27,040	Hypothetical Protein	-	76	KAK25_00029	100	PA25	YP_006299893.1
30	27,045	28,700	DEAD box helicase	-	551	DEAD box helicase	99	Ab26	YP_009044363.1
31	28,693	29,790	RecB exonuclease	-	365	RecB exonuclease	99	Ab26	YP_009044364.1
32	29,959	30,120	Hypothetical protein	-	53	KAK25_00032	100	PA25	YP_006299896.1
33	30,302	31,054	Hypothetical protein	-	251	Member of DUF669 phage protein family	99	PA25	YP_006299897.1
34	31,113	31,829	Rec A	-	238	RecA	100	Ab26	YP_009044366.1
35	31,884	32,324	Hypothetical protein	-	146	Hypothetical protein	99	Ab26	YP_009044367.1
36	32,401	32,958	MazG	-	185	MazG	98	Ab26	YP_009044368.1
37	33,078	33,284	Transcriptional regulator	+	68	Hypothetical protein	99	Ab26	YP_009044369.1
38	33,274	35,595	Replicative Primase/Helicase	+	773	Replicative primase/helicase	99	PA25	YP_006299902.1
39	35,741	36,193	Hypothetical protein	+	150	MAM_066	54	ΦMAM1^a^	YP_007349045.1
40	36,281	36,430	Hypothetical protein	+	49	KAK25_00040	69	PA25	YP_006299904.1
41	36,764	36,955	Hypothetical protein	+	63	ORF038	94	PA73	YP_001293445.1
42	36,952	37,248	Hypothetical protein	+	98	ORF039	100	PA73	YP_001293446.1
43	37,269	37,424	Hypothetical protein	+	59	Hypothetical protein	92	Ab26	YP_009044375.1
44	37,399	37,641	Hypothetical protein	+	80	ORF040	99	PA73	YP_001293447.1
45	37,748	37,945	Hypothetical protein	+	73	X805_23910	58	DSM 6575^b^	KDB52021.1
46	37,993	38,367	Hypothetical protein	+	124	KAK25_00046	97	PA25	YP_006299910.1
47	38,428	38,649	Hypothetical protein	+	73	KAK25_00047	99	PA25	YP_006299911.1
48	38,646	39,182	Vsr endonuclease	+	178	KAK25_00048	100	PA25	YP_006299912.1
49	39,170	39,385	Hypothetical protein	+	71	Hypothetical protein	100	Ab26	YP_009044380.1
50	39,382	39,561	Hypothetical protein	+	59	KAK25_00050	98	PA25	YP_006299914.1
51	39,622	39,927	Hypothetical protein	+	101	ORF045	100	PA73	YP_001293452.1
52	39,944	40,234	Hypothetical protein	+	96	ORF046	99	PA73	YP_001293453.1
53	40,227	40,460	Hypothetical protein	+	77	KAK25_00053	99	PA25	YP_006299917.1
54	40,531	40,698	Hypothetical protein	+	55	ORF048	100	PA73	YP_001293455.1
55	40,689	41,156	dCMP deaminase	+	145	dCMP deaminase	97	PA25	YP_009044383.1
56	41,162	41,545	Hypothetical protein	+	127	ORF050	100	PA73	YP_001293457.1
57	41,580	41,789	Hypothetical protein	+	69	ORF051	100	PA73	YP_001293458.1
58	41,873	42,445	Hypothetical protein	+	190	ORF052	99	PA73	YP_001293459.1

^a^ *Serratia* phage ΦMAM1

^b^ *Sphaerotilus natans* subsp. *natans* DSM 6575

### Analysis of modules

The proteins identified in DLP1 and DLP2 can be classified into three general categories: lysis, virion morphogenesis (including DNA packing and capsid/tail morphogenesis) and DNA replication/repair. The ORFs of DLP1 and DLP2 are syntenic, and the predicted proteins are similar with only a few variations from each other (Tables 3 and 4), yet these two phages exhibit two completely different plaque development characteristics (Fig. 1). It is also of interest to note that no genes encoding known or putative virulence factors were discovered in the genomes of phages DLP1 and DLP2, or any other related phages in this family.

#### Lysis

Genes putatively encoding the lysis proteins holin, lysin, Rz, Rz1 and a hypothetical protein have been identified in DLP1. A BLASTP search of predicted protein gp1 shows that it is similar to a phage protein family found in lysis cassettes that was identified in phage PA25. A BLASTP search also showed gp2 to be a putative holin protein similar to those identified in PA25 and PA73. Analysis of this gp2 protein with TMHMM revealed it has two transmembrane domains; thus, gp2 is a predicted to be a class II holin [40]. Gene gp3 is nearly identical to the endolysin of PA25. Gp4 and gp5 proteins are similar to the Rz protein of Ab26 and Rz1 of PA25 respectively. The Rz protein is a class II inner membrane protein with an N-terminal domain and Rz1 is a proline-rich outer membrane lipoprotein [41]. The Rz/Rz1 proteins contribute to lysis by fusing to the inner and outer membranes following holin and endolysin activity to facilitate phage release [42]. The gp4 protein is predicted to contain a single N-terminal transmembrane domain, a characteristic of Rz proteins [40, 43]. LipoP analysis of gp5 shows a signal peptidase II cleavage site between amino acids 20 and 21, resulting in a 73 amino acid protein with 7 proline residues (9.6 % proline) [44].

The lysis predicted proteins identified in DLP2 are similar to those also identified in phage DLP1. However, there are also some differences. The gp3 of DLP2 is most closely related to ORF003 of PA73 and also the endolysin protein in PA25. Like DLP1, gp4 of DLP2 is similar to the Rz protein of phage Ab26. Although DLP2 gp5 required manual annotation, BLASTP analysis showed it is most closely related to Rz1 of Ab26, rather than phage PA25. However, LipoP analysis revealed the identical signal peptidase II cleavage site as for phage DLP1 gp5. Analysis of the lysis module for DLP1 and DLP2 did not reveal why phage DLP1 exhibits delayed plaque development when compared to phage DLP2. One hypothesis is that gp32 of DLP1, encoding a hypothetical protein not found in DLP2 (most closely related to gp055 of *Erwinia* phage vB_EamP-S6), contributes to the delayed plaque development of DLP1. However, until a definitive function for the DLP1 gp32-encoded protein can be established, this hypothesis remains unproven.

#### Virion morphogenesis

As discussed above, DLP1 is closely related to phage DLP2, and phages PA25, Ab26 and PA73, whose proteins are generally uncharacterized. BLASTP analysis of the 20 genes involved in virion morphogenesis in DLP1 identified 13 genes with putative functions: two involved in DNA packaging, four involved in capsid morphogenesis and seven involved in tail morphogenesis. The DNA packaging proteins gp6 (small terminase subunit) and gp7 (large terminase subunit) are similar to the small terminase subunit of PA25 and large terminase subunit of Ab26 respectively. Protein gp8 shares a 98 % identity to the portal protein of Ab26. Although gp9 shares 100 % identity to uncharacterized ORF008 of PA73, it has been assigned a putative function due to its high similarity to the F-like head morphogenesis protein of Ab26 (Table 3). Gp10 shares 100 % identity to the scaffold protein of PA25. The gp11 is most similar to ORF010 of PA73, but its high similarity to the major capsid protein of Ab26 has allowed a putative function to be assigned to this protein (Table 3). The seven proteins involved in tail morphogenesis are gp15 (head-tail joining protein), gp16 (minor tail protein), gp17 (major tail protein), gp18 (tail chaperonin), gp19 (tail chaperonin), gp21 (tape measure protein) and gp26 (central tail hub). Both gp15, closely related to the virion protein of Ab26, and gp16, closely related to ORF015 of PA73, have been assigned putative functions due to their similarities to the head-tail joining protein and minor tail protein of *Burkholderia* phage KL1 respectively (Table 3) [33]. Protein gp17 shares a 99 % identity to the major tail protein of PA25. Gp17 is 100 % identical to ORF017 of PA73, but has been assigned the putative function of tail chaperonin due to its similarity to Ab26 tail chaperonin. Like gp17, gp18 is predicted to be a tail chaperonin protein, and has 100 % identity to the PA25 tail chaperonin protein. Both gp21 and gp26 are closely related to the tape measure protein and central tail hub of PA25, respectively. Analysis of DLP2 with BLASTP revealed the virion morphogenesis proteins are nearly identical to those of DLP1, with only minor differences (Table 4).

#### DNA replication and repair

DLP1 and DLP2 have seven and eight identified proteins, respectively, identified to be involved in DNA replication and repair at the same gene position: DNA polymerase (gp27), replicative clamp (gp28), RecB exonuclease (gp31 - DLP2 only), RecA (gp34), MazG (gp36), replicative primase/helicase (gp39), Vsr endonuclease (gp48) and dCMP deaminase (gp54 in DLP1, gp55 in DLP2) (Tables 3 and 4; Fig. 5). Three and two additional proteins were assigned putative functions following bioinformatics analysis, in DLP1 and DLP2, respectively, based on their high similarities to known proteins: DEAD box helicase (gp30), RecB exonuclease (gp31 - DLP1 only) and a transcriptional regulator (gp37) (Tables 3 and 4; Fig. 5).

BLASTP analysis for DLP1 and DLP2 gp27 shows it is 99 % identical to a putative DNA polymerase in PA25. The replicative clamp of PA25 shares a 99 % identity to gp28 of DLP1 and 95 % identity to gp28 of DLP2. In DLP1, gp31 is 100 % identical to ORF030 in PA73, though its putative function was assigned due to its 95 % identity to the RecB exonuclease of Ab26. Gp31 in DLP2 has 99 % identity to the RecB exonuclease of Ab26. HHpred analysis of the gp31 protein for DLP1 and DLP2 revealed the proteins are similar to the exonuclease of the λ Red recombination complex (DLP1: 99 %, E-value of 3.8e^−17^; DLP2: 99 %, E-value 5.6e^−17^) [45]. The RecA protein of Ab26 shares 99 % and 100 % identity to the gp34 proteins of DLP1 and DLP2, respectively. The protein gp37 of DLP1 (100 % identity to ORF035 of PA73) and DLP2 (99 % identity to hypothetical protein in Ab26) have been assigned the putative function of transcriptional regulator due to their similarity to the transcriptional regulator of the *Burkholderia* phage KL1 [33]. The protein gp38 for both DLP1 and DLP2 shares 99 % identity to the replicative primase/helicase found in PA25. Both DLP1 and DLP2 contain a Vsr endonuclease (gp48), although gp48 of DLP1 is most similar to KL1 Vsr endonuclease, whereas gp48 of DLP2 is most similar to the Vsr endonuclease encoded by Ab26 (Tables 3 and 4). The dCMP deaminase (gp54 of DLP1 and gp55 in DLP2) of both phages is most closely related to the dCMP deaminase of PA25 (98 and 97 % identity, respectively). Protein gp30 of DLP1 and DLP2 is 100 % identical to ORF029 of PA73, but a putative function has been assigned in both phages, as gp30 is 99 % identical to the DEAD box helicase protein of Ab26 for both phages. DEAD box helicases are vital in RNA metabolism, as they function to fold RNA molecules into their correct secondary structures and realign RNA-protein interactions with the use of ATP [46].

A predicted protein of interest in DLP1 and DLP2 is MazG, which is encoded by gp36 in DLP1 (89 % identity to MazG of PA25), and gp36 in DLP2 (98 % identity to MazG of Ab26). During times of stress in bacteria, the unusual nucleotides pppGpp and ppGpp begin to accumulate, synthesized by the proteins SpoT and RelA respectively [47]. Amino acid starvation activates RelA to synthesize ppGpp, whereas other bacterial stressors such as carbon or nitrogen starvation triggers SpoT to synthesize pppGpp [47, 48]. The pppGpp nucleotide can be converted into ppGpp through the enzyme GppA phosphatase [47]. Both of these unusual nucleotides are involved in the global response to stressful conditions within the bacteria, though ppGpp is a more potent regulatory nucleotide for growth inhibition [47, 49]. MazG fits into this regulatory pathway by depleting the accumulated ppGpp, thus reducing growth inhibition [50]. The action of phage-encoded MazG has been of interest to researchers, as many marine phages have been found to encode MazG homologs [51]. It has been speculated that phage-encoded MazG operates to reduce the ppGpp pool within stationary-phase infected cells [52], thus enhancing propagation of phage progeny in bacterial cells growing in nutrient limiting conditions. The host bacterium for DLP1 and DLP2, *S. maltophilia,* has been isolated from nutrient-limited environments, such as ultrapure and deionized water [2, 53]. The presence of MazG in DLP1 and DLP2 could potentially offer a competitive advantage over MazG-deficient phages replicating in stationary phase *S. maltophilia*.

### Phage relatedness

The two *S. maltophila* phages DLP1 and DLP2 differ from each other based upon RFLP analysis, DNA comparison analysis, protein:protein comparison analysis, the presence of insertions/deletions (indels), genetic synteny, as well as the phenotypic differences presented, which include different host ranges and the timing of plaque formation. Based upon these analyses, which include changes to both structural and replication genes and their predicted gene products, we conclude that they are significantly different enough in genetic content and biology to be considered individual phages and not merely variants of one another. There is sufficient genomic, proteomic and biological differences that, although they are related phages, DLP1 and DLP2 are not (or are no longer) close variants of each other. These differences include 1369 base pair changes and 157 gaps required to align the DNA, and three genomic locations where DLP1 and DLP2 have acquired completely different genes, which originate from entirely different sources (Additional file 2: Table S2). In DLP1, ORF 32 encodes a protein of 175 amino acids with no known homolog, whereas in DLP2, ORF 32 encodes a 53 amino acid protein that is homologous to a gene found in phage PA25. In DLP1, ORF 39 encodes a 66 amino acid protein without a homolog, whereas DLP2 encodes a protein 150 amino acids in length, also with without known homologs. In DLP2, ORF 55 encodes a 55 amino acid protein that has homologs in both PA25 and PA73, whereas DLP1 has no coding DNA in this region of its genome. Besides these obvious differences, and even though these phages exhibit high average identity across their entire genomes and share almost all proteins, it is still possible that the differences within protein sequences may be associated with the functional differences displayed during bacterial host infection. For example, there are proteins that differ significantly between DLP1 and DLP2 (i.e. a large number of gaps are required to complete alignment), such as ORFs 13, 21, 33, 48, 51 and 54 (encoding a hypothetical protein, a tail tape measure structural protein, a hypothetical protein, a VSR endonuclease replication protein, a hypothetical protein and a dCMP deaminase replication protein, respectively).

Similar to bacterial relatedness, we suggest that phage relatedness is an arbitrary ideal, and that there are no set guidelines as to what constitutes a phage variant versus what is a different but related phage. In order of relatedness, it is clear that DLP1 is most related to DLP2, and then in order of decreasing relatedness, *Pseudomonas* phages PA25, PA73, Ab26 and finally *Burkholderia* phage KL1 (Additional file 2: Table S2). Together, they are similar enough to be considered as a *Siphoviridae* sub-family, but how would one delineate them as variants of the same phage versus related phages of a continuum? For example, DLP2 is more related to PA25 circa ORF 32, and more related to PA25 and PA73 circa ORF 55, than DLP1, even though DLP2 shares the most homology with DLP1. Therefore, how much genetic, proteomic and biological differences must be evident before phages are separated into different “species”? Based upon the biological differences (including host range and plaque formation differences), the significant genetic alterations (including the presence of “indels”), and the protein level differences (highly variable but related protein sequences) presented, we conclude that each of these phages are different but related phages. These analyses confirm the idea that, although the phage genome DNA sequences are syntenic, significant changes have occurred between every member of this sub-family of phages, which is also reflected in the biological differences exhibited by phages DLP1 and DLP2.

## Conclusions

Although relatively rare, the incidence of phage broad host range specificity at the genera level is being increasingly studied in the food production industry, mainly with the *Salmonella* and *Escherichia* genera [54–57]. This study is the first to identify and characterize phages capable of infecting pathogenic bacteria across taxonomic orders. DLP1 and DLP2 are closely related phages that share a high similarity to *P. aeruginosa* phages vB_Pae-Kakheti25, vB_PaeS_SCH_Ab26, and PA73 and lesser similarity to *Burkholderia* phage KL1. Phage DLP1, possessing a 42,887 bp genome, is predicted to encode 57 proteins and exhibits a delayed plaque development phenotype. Unlike DLP1, phage DLP2 exhibits normal plaque development, but possesses a relatively similar genome 42,593 bp in length. The cause of the delayed plaque development in phage DLP1 is yet unknown, but genomic comparison suggests that gene variants encoded by or genes acquired by DLP1 may contribute to the observed lysis phenotype differences. The use of phage therapy may be one of the best treatment options for otherwise untreatable drug resistant bacterial infections [5, 11, 58]. The genomic characterization of broad-host range phages such as DLP1 and DLP2 is the first step towards developing an effective phage therapy strategy for *S. maltophilia*.

## Methods

### Bacterial strains and growth conditions

Five *S. maltophilia* and eight *P. aeruginosa* strains were acquired from the Canadian *Burkholderia cepacia* complex Research and Referral Repository (Vancouver, BC). The *S. maltophilia* strains used for isolation of phage from soil samples were D1585, D1571, D1614, D1576 and D1568. An additional 22 *S. maltophilia* strains were gifted from the The Provincial Laboratory for Public Health - North (Microbiology), Alberta Health Services, for host range analysis. All strains were grown aerobically overnight at 30 °C on half-strength Luria-Bertani (½ LB) solid medium or in ½ LB broth with shaking at 225 RPM.

### Phage isolation, propagation and electron microscopy

DLP1 and DLP2 were isolated from Red Deer River sediment and *Linum lewisii* (blue flax) soil, respectively, using standard extraction protocols [59]. Environmental samples were incubated with shaking at 30 °C in ½ LB broth, modified suspension medium (SM) (50 mM Tris–HCl [pH 7.5], 100 mM NaCl, 10 mM MgSO_4_), and *S. maltophlia* D1585 liquid culture. Solids were pelleted by centrifugation and the supernatant was filter-sterilized using a Millex-HA 0.45 μm syringe driven filter unit (Millipore, Billerica, MA). These were plated in soft agar overlays with strain D1585, and incubated overnight at 30 °C followed by observation for >24 h at room temperature. For each environmental sample, a single plaque was isolated using a sterile Pasteur pipette, suspended in 500 μl of modified SM with 20 μl chloroform and incubated 1 h at room temperature to generate stocks for DLP1 and DLP2.

Propagation of the phages was performed using soft agar overlays: 100 μl liquid culture and 100 μl phage stock were incubated 20 min at room temperature, mixed with 3 ml 0.7 % ½ LB top agar, overlaid on a plate of ½ LB solid medium, and incubated at 30 °C until plaque formation was complete. High titre stocks were made by overlaying plates with confluent lysis with 3 ml modified SM and incubated >1 h at room temperature on a platform rocker. The supernatant was recovered, pelleted by centrifugation for 5 min at 10,000 × g, filter-sterilized using a Millex-HA 0.45 μm syringe-driven filter unit (Millipore, Billerica, MA) and stored at 4 °C. Titre of stocks was obtained using serial dilutions of phage stock into SM, followed by soft agar overlay technique and incubation at 30 °C until plaque formation was complete.

For electron microscopy, phage stocks were prepared as described above with the following modifications: ½ LB agarose plates and ½ LB soft agarose were used for overlays, MilliQ-filtered water for phage recovery and a 0.22 μm filter was used for syringe-driven filtration. A carbon-coated copper grid was incubated with lysate for 2 min and stained with 4 % uranyl acetate for 30 s. Transmission electron micrographs were captured using a Philips/FEI (Morgagni) transmission electron microscope with charge-coupled device camera at 80 kV (University of Alberta Department of Biological Sciences Advanced Microscopy Facility). The capsid diameter and tail length was calculated using Microsoft Excel based on measurements from nine individual virions.

### Phage host range analysis, and PCR confirmation

Host range analysis was performed using a panel of 27 clinical *S. maltophilia* and 19 *P. aeruginosa* strains (Tables 1 and 2), and 25 other *Pseudomonas* and Gram-negative bacterial species (Additional file 1: Table S1). Soft-agar overlays containing 100 μl liquid bacterial culture were allowed to solidify for 10 min at room temperature. These plates were spotted with 10 μl drops of DLP1 or DLP2 at multiple dilutions and assayed for clearing (confluent phage lysis), and/or plaque formation after incubation for 36 h at 30 °C. If plaques were formed, a single plaque from a successful infection plate was picked to propagate as a working stock solution for further analysis. In order to confirm that the plaque contained DLP1 or DLP2 particles, PCR analysis was conducted on each purified phage solution using TopTaq DNA polymerase and buffers (Qiagen) and primers specific to each phage (DLP1F: ACACTGGCGAAGGATTACGG, DLP1R: GCCTTTCGAAATTCGCCGTT and DLP2F: CGGCTTTTTCGTGCCTGTAA, DLP2R:ACTCCTTTTCGATGCGTCCG) (Sigma-Genosys, Oakville, ON). These PCR products correspond to regions of DNA encoding part of ORF38, ORF39 and ORF40 in DLP1 and part of ORF38 and ORF39 in DLP2. PCR products were separated and visualized on a 1 % (*wt/vol*) agarose gel in 1x TAE (pH 8.0), and the product authenticity was confirmed by DNA sequencing. This test is a control experiment designed to ensure that the application of an exogenous phage does not induce a resident prophage into production. All samples that were positive for the production of phage clearing or plaques were subjected to confirmation of DLP1 or DLP2 phage production by PCR.

### Phage DNA isolation, RFLP analysis and sequencing

DLP1 and DLP2 genomic DNA was isolated from bacteriophage lysate using the Wizard Lambda DNA purification system (Promega Corp., Madison, WI) with a modified protocol [60, 61]. A 10 ml aliquot of filter-sterilized phage lysate (propagated on D1585 with agarose medium) was treated with 10 μl DNase I (Thermo Scientific, Waltham, MA), 100 μl 100x DNase I buffer (1 M Tris–HCl, 0.25 M MgCl_2_, 10 mM CaCl_2_), and 6 μl RNase (Thermo Scientific) and incubated 1 h at 37 °C to degrade the bacterial nucleic acids. Following incubation, 400 μl of 0.5 M EDTA and 25 μl of 20 mg/ml proteinase K (Applied Biosystems, Carlsbad, CA) were added and incubated 1 h at 55 °C to inactivate DNase I. After cooling to room temperature, the lysate was added to 8.4 g of guanidine thiocyanate, along with 1 ml of 37 °C resuspended Wizard DNA Clean-Up Resin (Promega Corporation, Madison, WI). This mixture was rocked for 10 min, and then pelleted by centrifugation at room temperature for 10 min at 5000 x g. The supernatant was drawn off until ~5 ml remained. This mixture was resuspended by swirling, transferred into a syringe attached to a Wizard Minicolumn (Promega Corporation), and pushed through the column. The column was then washed with 2 ml 80 % isopropanol and dried by centrifugation for 2 min at 10,000 x g. Phage DNA was eluted from the column following a 1 min incubation of 100 μl of 80 °C nuclease-free water (Integrated DNA Technologies, Coralville, IA) and centrifugation for 1 min at 10,000 x g. A NanoDrop ND-1000 spectrophotometer (Thermo Scientific, Waltham, MA) was used to determine purity and concentration of eluted DNA.

For each phage DNA sample, restriction fragment length polymorphism analysis was performed using three - 20 μl FastDigest EcoRI (Thermo Scientific) reactions containing l μg of phage DNA. Reactions were incubated at 37 °C for 5 min and separated on a 1 % (wt/vol) agarose gel in 1x TAE (pH 8.0). Preliminary sequencing of EcoRI phage DNA fragments cloned into pUC19 was performed as described previously [62, 63]. Phage DNA was submitted to The Applied Genomics Core at the University of Alberta for sequencing using MiSeq (Illumina, San Diego, CA) and assembled using the CLC Genomics Workbench (Qiagen, Toronto, ON). The genome sequences of DLP1 and DLP2 have been deposited in GenBank with the accession numbers KR537872 and KR537871, respectively.

### Bioinformatics analysis

Open reading frames (ORFs) for each contig were identified using the GLIMMER plugin [64] for Geneious [65] using the Bacteria and Archaea setting, as well as GeneMarkS (http://exon.gatech.edu/GeneMark/genemarks.cgi) for phage [66]. Conserved domain searches were performed using CD-Search [67]. The contigs were annotated using BLASTN and BLASTP (for full genomes and individual proteins, respectively) [68]. BLASTX and PHAST were used to search for similar sequences in the GenBank database. Sequence comparisons were visualized using Circos (http://circos.ca) [69] and NUCmer (http://mummer.sourceforge.net) [70] with the following parameters: breaklen = 200, maxgap = 90, mincluster = 65, minmatch = 20. Lysis protein analysis was performed using TMHMM for transmembrane region identification (http://www.cbs.dtu.dk/services/TMHMM/) [40].

### Availability of supporting data

Supporting data in the form of “Additional file 1: Table S1.” can be accessed through LabArchives, LLC at doi: 10.6070/H4CJ8BGT . Supporting data in the form of “Additional file 2: Table S2.” can be accessed through LabArchives, LLC at doi: 10.6070/H4H9936J .

## Additional files

Additional file 1: Table S1. Bacterial Species and Strains Not Sensitive to Phages DLP1 or DLP2. Twenty five different Pseudomonasand other Gram-negative bacteria were tested for sensitivity to phages DLP1 and DLP2 using high- and low-titre plaque overlay assays. None of the additional species or strains were observed to be infected or form plaques under the conditions tested. (DOCX 36 kb) Additional file 2: Table S2. Protein: protein comparison of the predicted proteins encoded by phages DLP1 (vB_SmaS-DLP_1), DLP2(vB_SmaS-DLP_2), vB_Pae-Kakheti25, vB_PaeS_SCH_Ab26, PA73, and KL1 (vB_BceS_KL1). Pairwise comparison was carried out using BLSATP analysis, and important relationships and descriptivecharacteristics were determined. (DOCX 70 kb)

## Abbreviations

ATP: Adenosine triphosphate
BLAST: Basic local alignment search tool
bp: Base pair
dCMP: Deoxycytidine monophosphate
DEAD: Asp-glu-ala-asp
DNA: Deoxyribonucleic acid
EDTA: Ethylenediaminetetraacetic acid
FDA: United States Food and Drug Administration
g: Gravitational force or grams
gp: Gene product
h: Hours
kb: Kilobases
kV: Kilovolts
LB: Luria-Bertani
M: Molar
min: Minutes
ml: Milliliters
mm: Millimeters
mM: Millimolar
nm: Nanometers
ORF: Open reading frame
PCR: Polymerase chain reaction
RNA: Ribonucleic acid
RPM: Rounds per minute
s: Seconds
SM: Suspension media
TAE: Tris base, acetic acid, EDTA
μl: Microliters
μm: Micrometers
vsr: Very short patch repair

## References

[1] Aarestrup FM, Aidara-Kane A, Sande-Bruinsma N van de, Falzon D, Grundmann H, et al. Antimicrobial resistance: global report on surveillance. World Health Organization; WHO Press, 2014:1–256.

[2] Brooke JS (2012). *Stenotrophomonas maltophilia*: an emerging global opportunistic pathogen. Clin Microbiol Rev.

[3] Waters V, Atenafu EG, Lu A, Yau Y, Tullis E, Ratjen F (2013). Chronic *Stenotrophomonas maltophilia* infection and mortality or lung transplantation in cystic fibrosis patients. J Cyst Fibros.

[4] Wainwright CE, France MW, O’Rourke P, Anuj S, Kidd TJ, Nissen MD, Sloots TP, Coulter C, Ristovski Z, Hargreaves M, Rose BR, Harbour C, Bell SC, Fennelly KP (2009). Cough-generated aerosols of *Pseudomonas aeruginosa* and other Gram-negative bacteria from patients with cystic fibrosis. BMJ.

[5] Kutter E, De Vos D, Gvasalia G, Alavidze Z, Gogokhia L, Kuhl S, Abedon ST (2010). Phage therapy in clinical practice: treatment of human infections. Curr Pharm Biotechnol.

[6] Semler DD, Lynch KH, Dennis JJ (2012). The promise of bacteriophage therapy for Burkholderia cepacia complex respiratory infections. Front Cell Infect Microbiol.

[7] Hoe S, Semler DD, Goudie AD, Lynch KH, Matinkhoo S, Finlay WH, Dennis JJ, Vehring R (2013). Respirable bacteriophages for the treatment of bacterial lung infections. J Aerosol Med Pulm Drug Deliv.

[8] Nobrega FL, Costa AR, Kluskens LD, Azeredo J (2015). Revisiting phage therapy: new applications for old resources. Trends Microbiol.

[9] Burrowes B, Harper DR, Anderson J, McConville M, Enright MC (2011). Bacteriophage therapy: potential uses in the control of antibiotic-resistant pathogens. Expert Rev Anti Infect Ther.

[10] Chanishvili N (2012). Phage therapy--history from Twort and d'Herelle through Soviet experience to current approaches. Adv Virus Res.

[11] Seed KD, Dennis JJ (2009). Experimental bacteriophage therapy increases survival of *Galleria mellonella* larvae infected with clinically relevant strains of the *Burkholderia cepacia* complex. Antimicrob Agents Chemother.

[12] Hagens S, Habel A, Ahsen U, von Gabain A (2004). Therapy of experimental *Pseudomonas* infections with a nonreplicating genetically modified phage. Antimicrob Agents Chemother.

[13] Morello E, Saussereau E, Maura D, Huerre M, Touqui L, Debarbieux L (2011). Pulmonary bacteriophage therapy on *Pseudomonas aeruginosa* cystic fibrosis strains: first steps towards treatment and prevention. PLoS One.

[14] Kumari S, Harjai K, Chhibber S (2011). Bacteriophage versus antimicrobial agents for the treatment of murine burn wound infection caused by *Klebsiella pneumoniae* B5055. J Med Microbiol.

[15] Waseh S, Hanifi-Moghaddam P, Coleman R, Masotti M, Ryan S, Foss M, MacKenzie R, Henry M, Szymanski CM, Tanha J (2010). Orally administered P22 phage tailspike protein reduces *Salmonella* colonization in chickens: prospects of a novel therapy against bacterial infections. PLoS One.

[16] Golshahi L, Lynch KH, Dennis JJ, Finlay WH (2011). In vitro lung delivery of bacteriophages KS4-M and Î¦KZ using dry powder inhalers for treatment of *Burkholderia cepacia* complex and *Pseudomonas aeruginosa* infections in cystic fibrosis. J Appl Microbiol.

[17] Semler DD, Goudie AD, Finlay WH, Dennis JJ (2014). Aerosol phage therapy efficacy in *Burkholderia cepacia* complex respiratory infections. Antimicrob Agents Chemother.

[18] Wright A, Hawkins CH, AnggÃ¥rd EE, Harper DR (2009). A controlled clinical trial of a therapeutic bacteriophage preparation in chronic otitis due to antibiotic-resistant *Pseudomonas aeruginosa*; a preliminary report of efficacy. Clin Otolaryngol.

[19] Merabishvili M, Pirnay J, Verbeken G, Chanishvili N, Tediashvili M, Lashkhi N, Glonti T, Krylov V, Mast J, Van Parys L, Lavigne R, Volckaert G, Mattheus W, Verween G, De Corte P, Rose T, Jennes S, Zizi M, De Vos D, Vaneechoutte M (2009). Quality-controlled small-scale production of a well- defined bacteriophage cocktail for use in human clinical trials. PLoS One.

[20] Rhoads DD, Wolcott RD, Kuskowski MA, Wolcott BM, Ward LS, Sulakvelidze A (2009). Bacteriophage therapy of venous leg ulcers in humans: results of a phase I safety trial. J Wound Care.

[21] Abedon ST, Kuhl SJ, Blasdel BG, Kutter EM (2011). Phage treatment of human infections. Bacteriophage.

[22] Chang HC, Chen CR, Lin JW, Shen GH, Chang KM, Tseng YH, Weng SF (2005). Isolation and characterization of novel giant *Stenotrophomonas maltophilia* phage phiSMA5. Appl Environ Microbiol.

[23] Hagemann M, Hasse D, Berg G (2006). Detection of a phage genome carrying a zonula occludens like toxin gene (zot) in clinical isolates of *Stenotrophomonas maltophilia*. Arch Microbiol.

[24] Chen CR, Lin CH, Lin JW, Chang CI, Tseng YH, Weng SF (2007). Characterization of a novel T4-type *Stenotrophomonas maltophilia* virulent phage Smp14. Arch Microbiol.

[25] Fan H, Huang Y, Mi Z, Yin X, Wang L, Fan H, Zhang Z, An X, Chen J, Tong Y (2012). Complete Genome Sequence of IME13, a *Stenotrophomonas maltophilia* bacteriophage with large burst size and unique plaque polymorphism. J Virol.

[26] Huang Y, Fan H, Pei G, Fan H, Zhang Z, An X, Mi Z, Shi T, Tong Y (2012). Complete genome sequence of IME15, the first T7-like bacteriophage lytic to pan-antibiotic-resistant *Stenotrophomonas maltophilia*. J Virol.

[27] Lee CN, Tseng TT, Chang HC, Lin JW, Weng SF (2014). Genomic sequence of temperate phage Smp131 of *Stenotrophomonas maltophilia* that has similar prophages in xanthomonads. BMC Microbiol.

[28] Liu J, Liu Q, Shen P, Huang YP (2012). Isolation and characterization of a novel filamentous phage from *Stenotrophomonas maltophilia*. Arch Virol.

[29] Petrova M, Shcherbatova N, Kurakov A, Mindlin S (2014). Genomic characterization and integrative properties of phiSMA6 and phiSMA7, two novel filamentous bacteriophages of *Stenotrophomonas maltophilia*. Arch Virol.

[30] GarcÃ­a P, MonjardÃ­n C, MartÃ­n R, Madera C, SoberÃ³n N, Garcia E, Meana A, SuÃ¡rez JE (2008). Isolation of new *Stenotrophomonas* bacteriophages and genomic characterization of temperate phage S1. Appl Environ Microbiol.

[31] Lacroix-Gueu P, Briandet R, LÃ©vÃªque-Fort S, Bellon-Fontaine MN, Fontaine-Aupart MP (2005). In situ measurements of viral particles diffusion inside mucoid biofilms. C R Biol.

[32] Briandet R, Lacroix-Gueu P, Renault M, Lecart S, Meylheuc T, Bidnenko E, Steenkeste K, Bellon-Fontaine MN, Fontaine-Aupart MP (2008). Fluorescence correlation spectroscopy to study diffusion and reaction of bacteriophages inside biofilms. Appl Environ Microbiol.

[33] Lynch KH, Stothard P, Dennis JJ (2012). Comparative analysis of two phenotypically-similar but genomically-distinct *Burkholderia cenocepacia*-specific bacteriophages. BMC Genomics.

[34] Sullivan MB, Waterbury JB, Chisholm SW (2003). Cyanophages infecting the oceanic cyanobacterium *Prochlorococcus*. Nature.

[35] Watkins SC, Smith JR, Hayes PK, Watts JEM (2014). Characterisation of host growth after infection with a broad-range freshwater cyanopodophage. PLoS One.

[36] Weitz JS, Poisot T, Meyer JR, Flores CO, Valverde S, Sullivan MB, Hochberg ME (2013). Phage-bacteria infection networks. Trends Microbiol.

[37] Karumidze N, Thomas JA, Kvatadze N, Goderdzishvili M, Hakala KW, Weintraub ST, Alavidze Z, Hardies SC (2012). Characterization of lytic *Pseudomonas aeruginosa* bacteriophages via biological properties and genomic sequences. Appl Microbiol Biotechnol.

[38] Kwan T, Liu J, Dubow M, Gros P, Pelletier J (2006). Comparative genomic analysis of 18 *Pseudomonas aeruginosa* bacteriophages. J Bacteriol.

[39] Pellegrin V, Juretschko S, Wagner M, Cottenceau G (1999). Morphological and biochemical properties of a *Sphaerotilus* sp. isolated from paper mill slimes. Appl Environ Microbiol.

[40] Krogh A, Larsson B, von Heijne G, Sonnhammer EL (2001). Predicting transmembrane protein topology with a hidden Markov model: application to complete genomes. J Mol Biol.

[41] Berry J, Summer EJ, Struck DK, Young R (2008). The final step in the phage infection cycle: the Rz and Rz1 lysis proteins link the inner and outer membranes. Mol Microbiol.

[42] Berry J, Savva C, Holzenburg A, Young R (2010). The lambda spanin components Rz and Rz1 undergo tertiary and quaternary rearrangements upon complex formation. Protein Sci.

[43] Summer EJ, Berry J, Tran TAT, Niu L, Struck DK, Young R (2007). Rz/Rz1 lysis gene equivalents in phages of Gram-negative hosts. J Mol Biol.

[44] Juncker AS, Willenbrock H, Von Heijne G, Brunak S, Nielsen H, Krogh A (2003). Prediction of lipoprotein signal peptides in Gram-negative bacteria. Protein Sci.

[45] Söding J, Biegert A, Lupas AN (2005). The HHpred interactive server for protein homology detection and structure prediction. Nucleic Acids Res.

[46] Owttrim GW (2013). RNA helicases: diverse roles in prokaryotic response to abiotic stress. RNA Biol.

[47] Mechold U, Potrykus K, Murphy H, Murakami KS, Cashel M (2013). Differential regulation by ppGpp versus pppGpp in *Escherichia coli*. Nucleic Acids Res.

[48] Magnusson LU, Farewell A, Nyström T (2005). ppGpp: A global regulator in *Escherichia coli*. Trends Microbiol.

[49] Maciag M, Kochanowska M, Lyzeń R, Wegrzyn G, Szalewska-Pałasz A (2010). ppGpp inhibits the activity of *Escherichia coli* DnaG primase. Plasmid.

[50] Gross M, Marianovsky I, Glaser G (2006). MazG -- a regulator of programmed cell death in *Escherichia coli*. Mol Microbiol.

[51] Bryan MJ, Burroughs NJ, Spence EM, Clokie MRJ, Mann NH, Bryan SJ (2008). Evidence for the intense exchange of MazG in marine cyanophages by horizontal gene transfer. PLoS One.

[52] Clokie MRJ, Mann NH (2006). Marine cyanophages and light. Environ Microbiol.

[53] Arvanitidou M, Vayona A, Spanakis N, Tsakris A (2003). Occurrence and antimicrobial resistance of Gram-negative bacteria isolated in haemodialysis water and dialysate of renal units: results of a Greek multicentre study. J Appl Microbiol.

[54] Matilla MA, Salmond GPC (2014). Bacteriophage $$ \boldsymbol{\Phi} $$MAM1, a viunalikevirus, is a broad-host-range, high-efficiency generalized transducer that infects environmental and clinical isolates of the enterobacterial genera *Serratia* and *Kluyvera*. Appl Environ Microbiol.

[55] Bielke L, Higgins S, Donoghue A, Donoghue D, Hargis BM (2007). *Salmonella* host range of bacteriophages that infect multiple genera. Poult Sci.

[56] Park M, Lee J-H, Shin H, Kim M, Choi J, Kang D-H, Heu S, Ryu S (2012). Characterization and comparative genomic analysis of a novel bacteriophage, SFP10, simultaneously inhibiting both *Salmonella enterica* and *Escherichia coli* O157:H7. Appl Environ Microbiol.

[57] Kim M, Ryu S (2011). Characterization of a T5-like coliphage, SPC35, and differential development of resistance to SPC35 in *Salmonella enterica* serovar typhimurium and *Escherichia coli*. Appl Environ Microbiol.

[58] Ryan EM, Alkawareek MY, Donnelly RF, Gilmore BF (2012). Synergistic phage-antibiotic combinations for the control of *Escherichia coli* biofilms in vitro. Immunol Med Microbiol.

[59] Seed KD, Dennis JJ (2005). Isolation and characterization of bacteriophages of the *Burkholderia cepacia* complex. FEMS Microbiol Lett.

[60] DNA isolation from Lambda lysates using the Wizard® DNA Clean-Up System. [http://www.promega.ca/resources/pubhub/enotes/dna-isolation-from-lambda-lysates-using-the-wizard-dna-cleanup-system/].

[61] Lynch KH, Abdu AH, Schobert M, Dennis JJ (2013). Genomic characterization of JG068, a novel virulent podovirus active against *Burkholderia cenocepacia*. BMC Genomics.

[62] Lynch KH, Seed KD, Stothard P, Dennis JJ (2010). Inactivation of *Burkholderia cepacia* complex phage KS9 gp41 identifies the phage repressor and generates lytic virions. J Virol.

[63] Lynch KH, Stothard P, Dennis JJ (2010). Genomic analysis and relatedness of P2-like phages of the *Burkholderia cepacia* complex. BMC Genomics.

[64] Delcher AL, Bratke KA, Powers EC, Salzberg SL (2007). Identifying bacterial genes and endosymbiont DNA with Glimmer. Bioinformatics.

[65] Drummond A, Ashton B, Buxton S, Cheung M, Cooper A, Duran C, Heled J, Kearse M, Markowitz S, Moir R, Stones-Havas S, Sturrock S, Swidan F, Thierer T, Wilson A (2013). Geneious.

[66] Besemer J, Lomsadze A, Borodovsky M (2001). GeneMarkS: a self-training method for prediction of gene starts in microbial genomes. Implications for finding sequence motifs in regulatory regions. Nucleic Acids Res.

[67] Marchler-Bauer A, Lu S, Anderson JB, Chitsaz F, Derbyshire MK, DeWeese-Scott C, Fong JH, Geer LY, Geer RC, Gonzales NR, Gwadz M, Hurwitz DI, Jackson JD, Ke Z, Lanczycki CJ, Lu F, Marchler GH, Mullokandov M, Omelchenko MV, Robertson CL, Song JS, Thanki N, Yamashita RA, Zhang D, Zhang N, Zheng C, Bryant SH (2011). CDD: a conserved domain database for the functional annotation of proteins. Nucleic Acids Res.

[68] Altschul S, Madden T (1997). Gapped BLAST and PSI-BLAST: a new generation of protein database search programs. Nucleic Acids.

[69] Krzywinski M, Schein J, Birol I, Connors J, Gascoyne R, Horsman D, Jones SJ, Marra MA (2009). Circos: An information aesthetic for comparative genomics. Genome Res.

[70] Delcher AL, Phillippy A, Carlton J, Salzberg SL (2002). Fast algorithms for large-scale genome alignment and comparison. Nucleic Acids Res.

