# Supplementary material for: The isolation and characterization of two Stenotrophomonas maltophilia bacteriophages capable of cross-taxonomic order infectivity
Source: BMC Genomics. 2015 Sep 3;16(1):664. doi: 10.1186/s12864-015-1848-y (PMC4559383; doi:10.1186/s12864-015-1848-y)
Supplement: Additional file 1: Table S1. — Bacterial Species and Strains Not Sensitive to Phages DLP1 or DLP2. Twenty five different Pseudomonasand other Gram-negative bacteria were tested for sensitivity to phages DLP1 and DLP2 using high- and low-titre plaque overlay assays. None of the additional species or strains were observed to be infected or form plaques under the conditions tested. (DOCX 36 kb) [file 12864_2015_1848_MOESM1_ESM.docx]

Additional file 1: Table S2.

| **DLP1** | | **DLP2** | | | | | **PA25** | | | | | **PA73** | | | | | **Ab26** | | | | | **KL1** | | | | |
| --- | --- | --- | --- | --- | --- | --- | --- | --- | --- | --- | --- | --- | --- | --- | --- | --- | --- | --- | --- | --- | --- | --- | --- | --- | --- | --- |
| Predicted Protein  ORF AA | | AA | ID | Sim | Expect | Gaps | AA | ID | Sim | Expect | Gaps | AA | ID | Sim | Expect | Gaps | AA | ID | Sim | Expect | Gaps | AA | ID | Sim | Expect | Gaps |
| 1 | 84 | 84 | 81/ 84 | 81/ 84 | 3e-57 | 0 | 84 | 83/ 84 | 83/ 84 | 4e-51 | 0 | 84 | 84/ 84 | 84/ 84 | 1e-51 | 0 |  |  |  |  |  | 88 | 65/ 84 | 77/ 84 | 3e-40 | 0 |
| 2 | 88 | 88 | 84/ 88 | 85/ 88 | 4e-60 | 0 | 88 | 87/ 88 | 87/ 88 | 5e-55 | 0 | 88 | 85/ 88 | 86/ 88 | 4e-53 | 0 | 88 | 81/ 88 | 84/ 88 | 4e-51 | 0 | 98 | 35/ 86 | 53/ 86 | 1e-16 | 0 |
| 3 | 181 | 181 | 178/181 | 179/181 | 1e-136 | 0 | 181 | 178/ 181 | 179/ 181 | 8e-129 | 0 | 181 | 178/ 181 | 179/ 181 | 2e-128 | 0 | 181 | 177/ 181 | 179/ 181 | 2e-127 | 0 |  |  |  |  |  |
| 4 | 101 | 101 | 100/101 | 101/101 | 2e-71 | 0 | 101 | 100/101 | 101/ 101 | 5e-63 | 0 | 101 | 99/ 101 | 101/101 | 1e-62 | 0 | 101 | 100/101 | 101/101 | 4e-63 | 0 | 105 | 43/ 105 | 61/ 105 | 5e-14 | 8 |
| 5 | 93 | 93 | 91/ 93 | 91/ 93 | 6e-67 | 0 | 93 | 93/ 93 | 93/ 93 | 3e-60 | 0 |  |  |  |  |  | 93 | 91/ 93 | 91/ 93 | 1e-58 | 0 | 87 | 37/ 72 | 43/ 72 | 2e-12 | 9 |
| 6 | 162 | 162 | 161/162 | 161/162 | 4e-121 | 0 | 162 | 161/162 | 161/ 162 | 6e-113 | 0 | 162 | 162/162 | 162/162 | 4e-114 | 0 | 162 | 161/162 | 161/162 | 6e-113 | 0 | 163 | 92/ 153 | 113/153 | 6e-55 | 2 |
| 7 | 531 | 531 | 524/531 | 528/531 | 0 | 0 | 531 | 529/531 | 530/531 | 0 | 0 | 531 | 529/531 | 530/531 | 0 | 0 | 531 | 525/531 | 528/531 | 0 | 0 | 546 | 433/513 | 467/513 | 0 | 3 |
| 8 | 501 | 501 | 484/499 | 490/499 | 0 | 0 | 501 | 495/501 | 496/501 | 0 | 0 | 501 | 488/501 | 493/501 | 0 | 0 | 510 | 488/500 | 493/500 | 0 | 0 | 498 | 417/486 | 443/486 | 0 | 0 |
| 9 | 364 | 364 | 362/364 | 364/364 | 0 | 0 | 364 | 359/364 | 364/364 | 0 | 0 | 364 | 364/364 | 364/364 | 0 | 0 | 364 | 363/364 | 364/364 | 0 | 0 | 371 | 268/365 | 314/365 | 0 | 1 |
| 10 | 239 | 239 | 235/239 | 237/239 | 3e-173 | 0 | 239 | 239/239 | 239/239 | 2e-168 | 0 | 239 | 238/239 | 238/239 | 1e-167 | 0 | 239 | 235/239 | 239/239 | 2e-166 | 0 | 235 | 192/235 | 214/235 | 8e-133 | 0 |
| 11 | 325 | 325 | 322/325 | 324/325 | 0 | 0 | 325 | 323/325 | 324/325 | 0 | 0 | 325 | 323/325 | 324/325 | 0 | 0 | 324 | 303/325 | 310/325 | 0 | 1 | 324 | 291/325 | 308/325 | 0 | 1 |
| 12 | 134 | 134 | 133/134 | 133/134 | 3e-94 | 0 | 134 | 134/134 | 134/134 | 1e-86 | 0 | 134 | 134/134 | 134/134 | 1e-86 | 0 | 130 | 128/134 | 129/134 | 4e-81 | 4 | 138 | 96/139 | 101/139 | 8e-49 | 6 |
| 13 | 133 | 123 | 93/ 133 | 106/133 | 5e-55 | 13 | 133 | 92/ 131 | 103/131 | 4e-45 | 13 | 123 | 92/ 131 | 103/131 | 2e-45 | 13 | 123 | 94/133 | 104/133 | 4e-47 | 13 | 125 | 88/134 | 100/134 | 1e-45 | 13 |
| 14 | 172 | 172 | 155/172 | 164/172 | 1e-117 | 0 | 172 | 155/172 | 164/172 | 2e-109 | 0 | 172 | 154/172 | 163/172 | 8e-109 | 0 | 172 | 159/172 | 164/172 | 1e-111 | 0 | 172 | 139/172 | 154/172 | 1e-98 | 0 |
| 15 | 126 | 126 | 120/126 | 122/126 | 1e-89 | 0 | 126 | 124/126 | 124/126 | 2e-84 | 0 | 126 | 123/126 | 123/126 | 1e-83 | 0 | 126 | 120/126 | 123/126 | 8e-82 | 0 | 124 | 73/124 | 89/124 | 2e-43 | 2 |
| 16 | 151 | 151 | 145/151 | 147/151 | 6e-111 | 0 | 151 | 146/151 | 147/151 | 5e-103 | 0 | 151 | 146/151 | 147/151 | 5e-103 | 0 | 151 | 146/151 | 147/151 | 6e-103 | 0 | 152 | 102/147 | 121/147 | 3e-71 | 0 |
| 17 | 511 | 511 | 507/511 | 508/511 | 0 | 0 | 511 | 508/511 | 509/511 | 0 | 0 | 511 | 508/511 | 510/511 | 0 | 0 | 511 | 491/511 | 497/511 | 0 | 0 | 517 | 403/514 | 442/514 | 0 | 5 |
| 18 | 142 | 142 | 140/142 | 141/142 | 7e-105 | 0 | 142 | 142/142 | 142/142 | 1e-97 | 0 | 142 | 142/142 | 142/142 | 1e-97 | 0 | 142 | 140/142 | 141/142 | 8e-97 | 0 | 142 | 112/142 | 129/142 | 6e-79 | 0 |
| 19 | 118 | 114 | 112/114 | 114/114 | 4e-84 | 0 | 264 | 117/118 | 118/118 | 1e-78 | 0 | 118 | 118/118 | 118/118 | 8e-80 | 0 | 118 | 117/118 | 118/118 | 2e-79 | 0 | 264 | 92/118 | 103/118 | 5e-52 | 0 |
| 20 | 144 | 144 | 144/144 | 144/144 | 3e-107 | 0 | 144 | 144/144 | 144/144 | 5e-99 | 0 | 144 | 144/144 | 144/144 | 5e-99 | 0 | 144 | 144/144 | 144/144 | 5e-99 | 0 | 144 | 115/144 | 128/144 | 4e-77 | 0 |
| 21 | 1175 | 1187 | 1063/ 1214 | 1085/ 1214 | 0 | 66 | 1204 | 1162/1204 | 1170/1204 | 0 | 29 | 1204 | 1161/1204 | 1171/1204 | 0 | 29 | 1204 | 1146/1204 | 1167/1204 | 0 | 29 | 1272 | 633/1117 | 764/1117 | 0 | 72 |
| 22 | 320 | 320 | 315/320 | 318/320 | 0 | 0 | 320 | 279/320 | 296/320 | 0 | 0 | 320 | 259/320 | 282/320 | 0 | 2 | 320 | 257/320 | 279/320 | 0 | 2 |  |  |  |  |  |
| 23 | 321 | 321 | 306/321 | 316/321 | 0 | 0 | 317 | 211/326 | 244/326 | 3e-132 | 14 | 303 | 178/322 | 221/322 | 3e-106 | 20 | 375 | 173/322 | 218/322 | 3e-102 | 20 |  |  |  |  |  |
| 24 | 570 | 570 | 555/570 | 559/570 | 0 | 0 | 571 | 548/571 | 558/571 | 0 | 1 | 568 | 500/570 | 527/570 | 0 | 2 | 568 | 500/570 | 525/570 | 0 | 2 | 563 | 179/504 | 281/504 | 9e-98 | 6 |
| 25 | 274 | 274 | 272/274 | 274/274 | 0 | 0 | 274 | 271/274 | 274/274 | 0 | 0 | 274 | 270/274 | 273/274 | 0 | 0 | 274 | 269/274 | 273/274 | 0 | 0 | 273 | 136/276 | 178/276 | 7e-85 | 7 |
| 26 | 813 | 813 | 799/813 | 805/813 | 0 | 0 | 813 | 801/813 | 805/813 | 0 | 0 | 813 | 809/813 | 810/813 | 0 | 0 | 813 | 796/813 | 805/813 | 0 | 0 | 803 | 382/812 | 512/812 | 0 | 13 |
| 27 | 683 | 683 | 677/683 | 681/683 | 0 | 0 | 683 | 680/683 | 681/683 | 0 | 0 | 683 | 680/683 | 682/683 | 0 | 0 | 683 | 677/683 | 681/683 | 0 | 0 | 689 | 570/683 | 623/689 | 0 | 7 |
| 28 | 380 | 380 | 368/380 | 374/380 | 0 | 0 | 380 | 379/380 | 379/380 | 0 | 0 | 380 | 378/380 | 379/380 | 0 | 0 | 387 | 375/380 | 378/380 | 0 | 0 | 386 | 288/389 | 325/389 | 0 | 16 |
| 29 | 118 | 76 | 70/ 71 | 70/ 71 | 4e-50 | 0 | 75 | 70/ 71 | 70/ 71 | 1e-41 | 0 | 118 | 115/118 | 116/118 | 7e-78 | 0 |  |  |  |  |  |  |  |  |  |  |
| 30 | 551 | 551 | 551/551 | 551/551 | 0 | 0 | 551 | 550/551 | 550/551 | 0 | 0 | 551 | 551/551 | 551/551 | 0 | 0 | 554 | 550/551 | 551/551 | 0 | 0 | 551 | 498/551 | 528/551 | 0 | 0 |
| 31 | 365 | 365 | 346/365 | 352/365 | 0 | 0 | 365 | 365/365 | 365/365 | 0 | 0 | 365 | 365/365 | 365/365 | 0 | 0 | 365 | 347/365 | 352/365 | 0 | 0 | 332 | 238/368 | 271/368 | 6e-167 | 0 |
| 32 | 175 |  |  |  |  |  |  |  |  |  |  |  |  |  |  |  |  |  |  |  |  |  |  |  |  |  |
|  |  | 53 (ORF32) |  |  |  |  | 53 | 53/ 53 | 53/ 53 | 4e-28 | 0 |  |  |  |  |  |  |  |  |  |  |  |  |  |  |  |
| 33 | 248 | 251 | 200/234 | 203/234 | 3e-103 | 24 | 248 | 246/248 | 248/248 | 2e-165 | 0 | 248 | 245/248 | 247/248 | 1e-164 | 0 | 235 | 131/132 | 132/132 | 5e-88 | 0 | 261 | 111/138 | 123/138 | 4e-74 | 4 |
| 34 | 238 | 238 | 237/238 | 238/238 | 0 | 0 | 238 | 237/238 | 238/238 | 2e-173 | 0 | 238 | 237/238 | 238/238 | 2e-173 | 0 | 238 | 237/238 | 238/238 | 3e-173 | 0 | 238 | 216/238 | 228/238 | 2e-159 | 0 |
| 35 | 146 | 146 | 143/146 | 145/146 | 2e-103 | 0 | 146 | 145/146 | 145/146 | 2e-96 | 0 | 146 | 145/146 | 145/146 | 2e-96 | 0 | 238 | 142/146 | 144/146 | 1e-94 | 0 | 169 | 73/ 146 | 86/ 146 | 6e-32 | 9 |
| 36 | 185 | 185 | 160/185 | 169/185 | 1e-118 | 0 | 185 | 165/185 | 173/185 | 7e-115 | 0 | 185 | 164/185 | 172/185 | 2e-113 | 0 | 185 | 159/185 | 169/185 | 7e-115 | 0 | 179 | 127/179 | 141/179 | 3e-75 | 1 |
| 37 | 68 | 68 | 68/ 68 | 68/ 68 | 1e-48 | 0 | 62 | 62/ 62 | 62/ 62 | 2e-35 | 0 | 62 | 62/ 62 | 62/ 62 | 2e-35 | 0 | 68 | 67/ 68 | 68/ 68 | 2e-39 | 0 | 62 | 31/ 61 | 42/ 61 | 6e-13 | 0 |
| 38 | 773 | 773 | 763/773 | 769/773 | 0 | 0 | 773 | 770/773 | 771/773 | 0 | 0 | 773 | 769/773 | 770/773 | 0 | 0 | 780 | 763/773 | 769/773 | 0 | 0 | 769 | 656/773 | 709/773 | 0 | 4 |
| 39 | 66 NH |  |  |  |  |  |  |  |  |  |  |  |  |  |  |  |  |  |  |  |  |  |  |  |  |  |
|  |  | 150 (ORF39) | NH | NH | NH | NH |  |  |  |  |  |  |  |  |  |  |  |  |  |  |  |  |  |  |  |  |
| 40 | 49 | 49 | 38/ 49 | 43/ 49 | 5e-28 | 0 |  |  |  |  |  |  |  |  |  |  |  |  |  |  |  |  |  |  |  |  |
| 41 | 59 | 63 | 58/ 59 | 59/ 59 | 5e-41 | 0 |  |  |  |  |  | 63 | 55/ 59 | 57/ 59 | 5e-29 | 0 |  |  |  |  |  |  |  |  |  |  |
| 42 | 98 | 98 | 96/ 98 | 96/ 98 | 8e-72 | 0 | 91 | 25/ 60 | 38/ 60 | 6e-08 | 3 | 98 | 96/ 98 | 96/ 98 | 2e-63 | 0 | 91 | 25/ 60 | 38/ 60 | 6e-08 | 3 | 98 | 68/ 97 | 80/ 97 | 5e-43 | 0 |
| 43 | 59 | 59 | 52/ 59 | 57/ 59 | 1e-37 | 0 | 55 | 51/ 55 | 54/ 55 | 3e-28 | 0 |  |  |  |  |  | 67 | 53/ 59 | 55/ 59 | 2e-29 | 0 |  |  |  |  |  |
| 44 | 80 | 80 | 78/ 80 | 78/ 80 | 4e-57 | 0 | 80 | 76/ 80 | 77/ 80 | 4e-47 | 0 | 80 | 77/ 80 | 78/ 80 | 3e-48 | 0 | 80 | 77/ 80 | 78/ 80 | 3e-48 | 0 | 78 | 39/ 80 | 51/ 80 | 2e-14 | 2 |
| 45 | 73 | 73 | 73/ 73 | 73/ 73 | 5e-51 | 0 |  |  |  |  |  |  |  |  |  |  |  |  |  |  |  |  |  |  |  |  |
| 46 | 124 | 124 | 121/124 | 121/124 | 5e-85 | 0 | 124 | 123/124 | 124/124 | 7e-79 | 0 | 124 | 120/124 | 121/124 | 1e-69 | 0 | 124 | 117/124 | 120/124 | 1e-66 | 0 | 133 | 50/ 123 | 73/ 123 | 4e-10 | 3 |
| 47 | 73 | 73 | 72/ 73 | 72/ 73 | 3e-51 | 0 | 73 | 71/ 73 | 72/ 73 | 1e-42 | 0 |  |  |  |  |  | 74 | 53/ 73 | 60/ 73 | 1e-28 | 1 | 162 | 125/161 | 134/161 | 1e-83 | 1 |
| 48 | 163 | 178 | 135/181 | 140/181 | 3e-91 | 21 | 178 | 135/181 | 140/181 | 4e-83 | 21 | 179 | 135/181 | 140/181 | 4e-83 | 21 | 181 | 139/181 | 146/181 | 6e-92 | 18 | 162 | 125/161 | 134/161 | 1e-83 | 1 |
| 49 | 71 | 71 | 70/ 70 | 70/ 70 | 9e-51 | 0 | 67 | 67/ 67 | 67/ 67 | 8e-40 | 0 |  |  |  |  |  | 131 | 70/ 71 | 71/ 71 | 1e-42 | 0 | 66 | 47/ 62 | 54/ 62 | 4e-27 | 0 |
| 50 | 59 | 59 | 57/ 59 | 57/ 59 | 4e-41 | 0 | 59 | 56/ 59 | 56/ 59 | 2e-31 | 0 |  |  |  |  |  |  |  |  |  |  | 62 | 25/ 56 | 35/ 56 | .006 | 5 |
| 51 | 99 | 101 | 82/ 101 | 88/ 101 | 8e-55 | 2 | 101 | 82/ 101 | 87/ 101 | 6e-46 | 2 | 101 | 82/ 101 | 88/ 101 | 2e-46 | 2 |  |  |  |  |  |  |  |  |  |  |
| 52 | 96 | 96 | 94/ 96 | 96/ 96 | 5e-67 | 0 | 96 | 95/ 96 | 96/ 96 | 3e-59 | 0 | 96 | 95/ 96 | 96/ 96 | 3e-59 | 0 | 126 | 93/ 96 | 94/ 96 | 4e-58 | 0 |  |  |  |  |  |
| 53 | 77 | 77 | 76/ 77 | 77/ 77 | 8e-58 | 0 | 77 | 77/ 77 | 77/ 77 | 2e-49 | 0 | 77 | 76/ 77 | 77/ 77 | 8e-49 | 0 | 80 | 75/ 77 | 77/ 77 | 2e-48 | 0 | 78 | 50/ 76 | 62/ 76 | 2e-32 | 0 |
| 54 | 155 | 145 | 139/145 | 141/145 | 5e-106 | 0 | 155 | 152/155 | 153/155 | 4e-107 | 0 | 155 | 149/155 | 151/155 | 1e-105 | 0 | 155 | 150/155 | 151/155 | 3e-105 | 0 | 161 | 112/155 | 121/155 | 4e-73 | 3 |
|  |  | 55 (ORF 55) |  |  |  |  | 55 | 51/ 54 | 52/ 54 | 8e-11 | 0 | 55 | 54/ 54 | 54/ 54 | 7e-13 | 0 |  |  |  |  |  |  |  |  |  |  |
| 55 | 127 | 127 (ORF 56) | 127/127 | 127/127 | 5e-95 | 0 | 127 | 125/127 | 127/127 | 7e-86 | 0 | 127 | 127/127 | 127/127 | 9e-87 | 0 | 129 | 89/ 89 | 89/ 89 | 2e-57 | 0 | 124 | 54/ 127 | 76/ 127 | 8e-19 | 10 |
| 56 | 69 | 69 (ORF 57) | 66/ 69 | 66/ 69 | 8e-49 | 0 | 69 | 65/ 69 | 66/ 69 | 7e-40 | 0 | 94 | 66/ 69 | 66/ 69 | 7e-41 | 0 | 94 | 65/ 69 | 66/ 69 | 2e-40 | 0 | 68 | 29/ 69 | 45/ 69 | 1e-14 | 1 |
| 57 | 190 | 190 (ORF 58) | 187/190 | 189/190 | 1e-137 | 0 | 190 | 189/190 | 190/190 | 3e-131 | 0 | 190 | 189/190 | 190/190 | 3e-131 | 0 | 190 | 182/190 | 185/190 | 2e-125 | 0 | 184 | 124/161 | 185/161 | 2e-61 | 2 |

Abbreviations: ORF – Open Reading Frame; AA – amino acid; ID – AA Identity; Sim – AA Similarity; Expect – Expected number of chance matches in a random model; Gaps – Number of gaps added to increase alignment; NH – No available homolog. Dark gray shading shows phage genomes without homolog.
