# Supplementary material for: The isolation and characterization of two Stenotrophomonas maltophilia bacteriophages capable of cross-taxonomic order infectivity
Source: BMC Genomics. 2015 Sep 3;16(1):664. doi: 10.1186/s12864-015-1848-y (PMC4559383; doi:10.1186/s12864-015-1848-y)
Supplement: Additional file 2: Table S2. — Protein: protein comparison of the predicted proteins encoded by phages DLP1 (vB_SmaS-DLP_1), DLP2(vB_SmaS-DLP_2), vB_Pae-Kakheti25, vB_PaeS_SCH_Ab26, PA73, and KL1 (vB_BceS_KL1). Pairwise comparison was carried out using BLSATP analysis, and important relationships and descriptivecharacteristics were determined. (DOCX 70 kb) [file 12864_2015_1848_MOESM2_ESM.docx]

Additional file 2: Table S1 Bacterial Species and Strains Not Sensitive to Phages DLP1 or DLP2.

|  |  |  | **Phage** | |
| --- | --- | --- | --- | --- |
| **Species** | | **Strain** | **DLP1** | **DLP2** |
| *Pseudomonas fluorescens* | | D1492 | **−** | **−** |
|  |  | D1499 | **−** | **−** |
|  |  | D1557 | **−** | **−** |
|  |  | D1612 | **−** | **−** |
| *Pseudomonas putida* | | D0034 | **−** | **−** |
|  |  | D1275 | **−** | **−** |
|  |  | D1500 | **−** | **−** |
|  |  | D1501 | **−** | **−** |
| *Pseudomonas stutzeri* | | C9295 | **−** | **−** |
|  |  | D0399 | **−** | **−** |
|  |  | D0997 | **−** | **−** |
|  |  | D1035 | **−** | **−** |
| *Burkholderia multivorans* | | ATCC17616 | **−** | **−** |
|  |  | KLB | **−** | **−** |
| *Burkholderia cepacia* | | C6433 | **−** | **−** |
|  |  | C5393 | **−** | **−** |
|  |  | K56-2 | **−** | **−** |
| *Burkholderia gladioli* | | CEP0029 | **−** | **−** |
|  |  | CEP0071 | **−** | **−** |
|  |  | CEP0082 | **−** | **−** |
| *Acinetobacter baumannii* | | 17978 | **−** | **−** |
|  |  | 19606 | **−** | **−** |
|  |  | AYE | **−** | **−** |
|  |  | SDF | **−** | **−** |
|  |  | 1441-1 | **−** | **−** |
